# Rethinking pathways to well-being: the function of faith practice in distress alleviation among displaced Muslim women affected by war

**DOI:** 10.3389/fpsyt.2025.1335640

**Published:** 2025-07-21

**Authors:** Kathleen Rutledge

**Affiliations:** Queen Margaret University, Institute for Global Health and Development, Edinburgh, United Kingdom

**Keywords:** religious coping, displaced, Muslim, gender, mental health, faith, MHPSS, conflict

## Abstract

**Background:**

For many populations globally, coping approaches employed during times of extreme adversity are rooted in religious convictions. Positive religious coping following potentially traumatic events and in times of crisis is widely evidenced as resilience promoting. Despite international mandates for aid and mental health responses to enable such coping, there is limited guidance for work with distinct faith groups and limited quantitative evidence overall. This mixed methods study examined the role of faith in mental health among displaced Muslim women affected by conflict, highlighting implications for responders.

**Methods:**

A total of 160 questionnaires, 50 interviews, and four focus groups were conducted among 160 Sunni Muslim women in an Iraqi internally displaced persons (IDP) camp with subjects who had been affected by the Islamic State of Iraq and Syria (ISIS) conflict. A total of 19 faith leaders, MHPSS providers, and humanitarian workers were interviewed as key informants. Qualitative responses were analyzed using inductive thematic analysis, while statistical tests examined variable correlations between the mean scores of response groups.

**Results:**

Religious meanings were attributed to every aspect of daily life, in addition to shaping fundamental understandings of wellbeing, the ultimate goals of life, and the coping strategies employed. Religiosity was high. Prayer, reciting, or reading the Qur’an, and fasting were widely reported as a means of comfort, stress relief, divine protection, and daily provision. The function of faith practices in distress alleviation was mediated by the individual’s beliefs regarding the afterlife and by their perception of God’s “care” for their life and situation. Self-appraised “inadequate” faith practice—seen as incompatible with the fundamental goal of life for many in the study, entering Paradise after death—and feeling that God does not “care”, were variables associated with higher distress and poor mental health. Gender-blind approaches in the camp and exclusion of faith needs from assessments and response actions compounded distress by creating access barriers. Ensuring access to gender- and faith-sensitive coping resources (when requested by the affected individuals) is likely to boost mental health outcomes, particularly when such supports align with recovery and/or strengthening of the individual’s sense of connectedness to a benevolent, responsive God.

## 1 Introduction

In the wake of potentially traumatic events, such as exposure to conflict, natural calamities, violence, and forced displacement, many populations globally cope with and make sense of their suffering through belief systems and practices rooted in religious faith (1–3). Four out of five persons worldwide claim affiliation with a religious or faith tradition, with that figure projected to rise (4, 5).

For those affected by crisis and displacement, religion can be a “complex source of resilience,” facilitating a sense of identity and connection and serving as a source of psychosocial, spiritual, and social support on an individual and communal basis (1, 6, 7, p. 34). Participation in a religious community, in shared rituals and in calendrical rites, fasts, and festivals, can serve as a source of support and comfort and can provide a sense of identity through simultaneous connection to transnational and local communities of faith (1, 8–10). Individual extrinsic behaviors or rituals, such as praying and reading sacred texts, are frequently cited as critical resources for coping with hardship and managing the stressors of displacement (6, 11–13).

For many individuals who claim a faith affiliation, prayer, and related practices are seen as rituals of value because they are believed to facilitate a communion or relationship with a divine, transcendent power (6, 14, 15). The nature of that relationship—evidenced in part in the individual’s beliefs regarding the care, responsiveness, and benevolence of that power—is directly linked to the ultimate impact of those faith practices on psychological distress.

A wide body of evidence has shown that faith practice that reflects a “secure attachment” to the divine, meaning that the divine force is seen by the believer to be accessible, responsive, comforting, and protective, is associated with lower anxiety, decreased depression, and improved life satisfaction (16–19). This is often referred to as positive religious coping (20). Conversely, if a believer views the transcendent being as harsh, inaccessible, and inconsistent, this has been shown to exacerbate the adverse effects of stress and is linked to increased anxiety, depression, angry feelings, and increased callousness toward others (20–22). Studies solely among Muslim populations have shown similar results (1, 23, 24).

After experiencing potentially traumatic events or in the face of prolonged adversities, such as protracted displacement, underlying belief systems and fundamental assumptions about God, oneself, and the nature of the world may be severely tested (3, 22, 25, 26). Among populations of faith, religious belief systems often provide a framework through which adverse events are interpreted (2, 26, 27). This framework can be a source of “meaning, control, comfort, intimacy, and life transformation” in day-to-day life (18, 20, p. 54, 28). When significant adverse life events occur, however, assumptions about the world—that the world is meaningful and ordered, that people get what they deserve, and that the world is generally benevolent, being directed by a benevolent God—may be severely tested (22, 27).

Depending on the severity of the impact of the adversities faced and the level of dissonance between the earlier assumptions and present situation, distress may be severe. To reduce that distress, those affected must “reappraise” either the meaning they have given to the situation or alter their fundamental understanding of the world (1, 3, 25). The capacity of the individual to reconstrue, or reappraise, suffering “positively” has also been shown to buffer against the negative effects of trauma and promote improvements in physical and mental health during recovery (1, 21, 22, 29, 30).

For persons of faith that struggle to “reappraise” acute distress events “positively” and for whom the dissonance between their former understanding of God and the world and their current realities persists in causing acute distress, this may result in long-term, debilitating health and psychological consequences if unresolved (27, 31–34). In such cases, mental health and psychosocial support (MHPSS) personnel with literacy regarding the faith beliefs and needs of affected individuals are required (35–37).

To explore how religious beliefs and practices are engaged in coping and reappraisal, however, it is important to underscore that the definitions of suffering, wellbeing, and resilience are rooted deeply in culture (1, 30, 38). Rather than conceptualizing wellbeing and resilience as “merely the absence of mental problems” following “exposure to trauma” as is the case in many mental health programs in humanitarian contexts, wellbeing is widely defined around the world by cultural values (38, p. 393). These values include elements such as religious faith, good morals, family unity and harmony, social respectability and honor, service to family and community and perseverance or effort, collective unity, and/or environmental health (38–40).

Among persons of faith in Islamic contexts, foundational understandings of what suffering and wellbeing entail are deeply intertwined with religious beliefs (41–43). Concepts of suffering and wellbeing are grounded in a worldview that sees the individual created by God and linked to every other creature created by God (including spirits) (42). Wellbeing in an Islamic worldview is thus defined by one’s relationship with God and others, including complete surrender of the individual to the strength and perfection of God as experienced through life events that happen according the pre-ordained will of God (41–43).

The priority concern for many Muslims, however, is achieving *eternal* wellbeing, or union with Allah in Paradise for eternity after death (28, 43–45). The pathway to achieving eternal wellbeing set forth in Islam through surrender (the literal meaning of the word “Islam”) and living life according to the teachings captured in the sacred texts and Sharia law (41, 43).

It is the devout Muslim’s self-assessment of their degree of fulfillment of the requirements of the faith—and the implications of that assessment in relation to whether or not they will receive the reward of Paradise—that is strongly tied to distress and mental health outcomes.

The obligatory or required practices of the faith include the “five pillars.”^1^ According to Islamic teaching, on the Day of Judgment, Muslims will be held accountable for the degree to which they fulfilled these practices and for the actions they took in life to organize their character, behavior, and intentions according to the law, as those actions are the manifestations of love of Allah (28, 44, 45). The punishment meted out on the Day of Judgment will vary in degree of severity and duration according to one’s deeds in this life, with the worst punishment being the permanent separation from God and torment in hell and the greatest reward for Muslims being Paradise for eternity with Allah (46).

In addition to required Islamic duties, there are Sunnah practices that are voluntary and can, according to Islamic teaching, play a pivotal role enabling a believer to enter into Paradise. Sunnah, which are sayings, practices, and personal characteristics of the Prophet Mohammed, are seen as the normative example of how Muslims should strive to live (49). Sunnah relate to all aspects of life, from religious practices such as prayer and fasting to every day decisions such as what to wear, hygiene, treatment of others, control of infections, and dealing with anger (50–53). While Sunnah practices are voluntary, meaning there will be no punishment on the Day of Judgment if one does not do them, doing Sunnah is believed to enable reward in the afterlife, in addition to the perceived intrinsic reward in this life of following the example of Prophet Mohammed (53, 54). It is also believed that doing certain Sunnah practices, such as Sunnah prayers, may make up for any missing “obligatory” (*fard*) prayers (such as the five daily prayers) on Judgment Day (46).

Thus, for many Muslims, with the ultimate goal of entering Paradise with Allah for eternity in mind, self-appraisals of the degree of fulfillment of both the “required” and “voluntary” faith practices can become critical sources of comfort and reassurance or distress and anxiety. Faith practices are also seen as the primary means of facilitating a sense of connectedness and nearness to Allah now (43, 55), with perceptions of closeness to Allah shown to be associated with improved perceptions of wellbeing and coping capacity (56, 57). These faith-infused understandings of wellbeing now and after death are widely held in the Islamic world, including among many Muslim women from Iraq, the target group for this research (11, 58, 59).

The differences between the understandings of wellbeing and the pathways to distress alleviation between Islamic approaches and Global North psycho-therapeutic foundations are notable. As clinicians have sought to care for Muslims with mental illness, Islamic-influenced counseling methods and therapeutic interventions have been increasingly trialed and documented (44, 60–64). These models remain largely segregated to use in clinical contexts outside of the aid and development spheres, however. More broadly, international guidance from the United Nations and other industry leaders advising practitioners to seek to understand the explanatory models of distress in the local context and identify and engage “spiritual supports” (35, p. 106) when desired by the population often goes unheeded (37, 65). As a result, standard MHPSS approaches utilized among Muslim communities in displacement contexts frequently remain disconnected from the worldview foundations of many in the populations that they serve (66–68).

Research indicates that limited understanding of underlying belief systems and practices can lead to insufficiently adapted interventions that do not fully connect with the core drivers of distress or with the existing coping capacities of the individual, thus diminishing the effectiveness of interventions (69–71).

The way in which gender and faith identity interact to create barriers to access for women in many displacement contexts is also poorly understood, with limited adaptations made in practice to remove those barriers. While extensive studies have shown that mental health needs and coping capacities are often gendered (11–13, 72), fewer studies have explored the way in which religious identity and gender interact to diminish access to support services and faith-related coping resources. Those that have focused on this have identified a pattern of increased distress and vulnerability among displaced Muslim women resulting from barriers to protection services, mental health support, and aid available in mixed-gender public spaces or through male leaders only (73–77).

**Figure 1:**
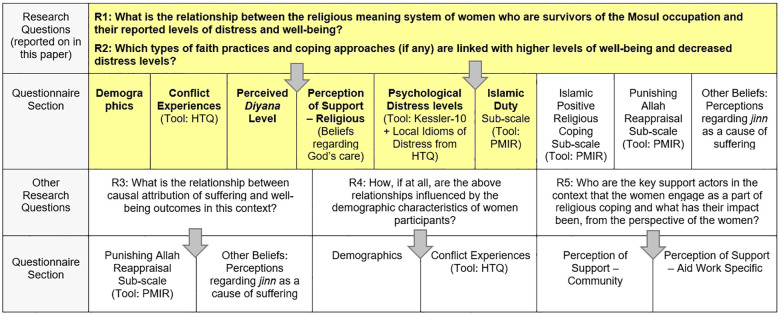
Mapping of research questions to selected tools and questionnaire sections (Highlighted cells: data reported in this paper.

This mixed-methods study sought to deepen the qualitative and quantitative evidence available on religious coping and mental health among displaced and conflict-affected Muslim women and to provide guidance that incorporates MHPSS best practices from emergency settings along with mental healthcare guidance informed by Islamic perspectives. The wider research study focused on five research questions (see **Figure 1**); this article presents results pertaining to two of the primary research questions, based on research conducted in Iraq between April and June 2019 among Sunni Muslim women from the Mosul region who suffered due to the ISIS conflict and were displaced in a camp for internally displaced persons (IDPs). The research questions of focus in this article include the following: 1) what is the relationship between the religious meaning system of women who are survivors of the Mosul occupation^2^ and their reported levels of distress and well-being? 2) Which types of faith practices and coping approaches (if any) are linked with higher levels of well-being and decreased distress levels?^3^.

## 2 Context

The self-titled Islamic State of Iraq and Syria (ISIS) emerged in northern Iraq in late 2013, occupying the Mosul region and other parts of the country from 2014 until 2017 (78, 79). ISIS brought with them a harsh regime of religious and social oppression, genocide, and violence, in the name of re-establishing a caliphate where a strict version of Sunni Islam would be lived (79–83). More than 2.1 million children, men, and women were displaced in 2014 alone, and hundreds of thousands of people, including Muslims, Yezidis, and Christians, suffered acutely (84–90). Systematic rape, public executions, and severe punishment for infractions were the norm, and there were chronic shortages of basic supplies in ISIS-controlled areas (80, 86–89, 91–93).

It is estimated that 1.5 million children, men, and women, most of whom were Sunni Muslim, remained in Mosul, Iraq’s second largest city, during the ISIS occupation (78, 87, 94). ISIS carried out brutal punishments and executed thousands of fellow Sunni Muslims in Mosul for actions such as wearing Western clothes, shaving one’s beard, and selling alcohol or drugs (80, 81, 83). In the detailed accounts captured by the *Mosul Eye* during the occupation, 455 executions, 118 whippings, 118 arrests, and 88 hand amputations were carried about by ISIS for violations of their policies in August and September 2015 alone (92). The majority of the victims were Sunni Muslims.

There was also a catastrophic loss of life among civilians as Iraqi armed forces sought to oust the extremist group, particularly during battles to retake major cities like Mosul (95–98). Although estimates of civilian deaths in the 9-month battle for Mosul (October 2016–July 2017) remain disputed, sources project that between 6,000 and 10,000 civilians lost their lives (95, 96).

More than 830,000 children, women, and men fled Mosul during the battle, and at the peak of displacement, following the recapture of the city in 2017, there were more than 3.3 million people displaced across Iraq as a result of the 3-year ISIS conflict (99, 100). Nearly 2 years later, at the start of this research in April 2019, approximately 1.7 million people remained displaced across the country, with more than 440,000 of these children, men, and women living in camps for internally displaced persons (IDPs; 101,102). In the Ninewa Governorate alone, where Mosul is situated, there were 218,000 children, men, and women living in 19 camps, including the camp selected for this study (101, 102).

As of 2019, the conditions in the camp selected for this study were harsh and deteriorating, with tents molding, reduction in food quantities per household taking effect despite no corresponding decrease in family size, limited electricity due to fuel cuts to the camp, agencies closing programs (including MHPSS and the only gender-based violence support program), very few income opportunities, restrictions on leaving the camp, no shade or other means of escaping the summer heat, and inter-governmental land disputes (Iraq-Kurdish Regional Government), insecurity, devastated infrastructure, and poverty preventing families from returning to their pre-displacement homes (103–106).

## 3 Materials and methods

### 3.1 Study sample

This mixed methods study was conducted among 160 Sunni Muslim women aged 18–64 years old who were living in a displacement camp in the Mosul region of Northern Iraq at the time of the study in 2019. All of the study participants had been impacted, through displacement, living under occupation, and/or direct exposure to combat, by the conflict with ISIS in Northern Iraq (2014–2017). The study aimed to explore the relationship (if any) between the religious meaning system and faith practices of the women in the study with psychological distress levels and perceived wellbeing.

The camp selected for this study, in the Ninewa governorate, was opened in November 2016 to receive those displaced during the battle for Mosul (see *Context*). The camp is not being named to protect the anonymity of study participants. The camp was selected in consultation with research host agencies, with accessibility being a key factor in terms of security and the ability to secure required checkpoint clearances and camp permissions to conduct the research.

At the start of the study in April 2019, there were 5,729 children, men, and women in 1,219 households in the camp, including 1,397 women aged 18 and over, according to camp management data. Nearly 100% of the camp population were Arab Sunnis (defined as adherents of Sunni Islam), with a small minority of Turkmen Sunnis, an ethnic group who consider themselves descendants of the Seljuq Turks (88). The selection criteria included women 18–65 years of age from the greater Mosul region who lived in this region at the time of the ISIS invasion and/or during the conflict between 2014 and 2017.

Study participants were recruited through random and purposive sampling. Sampling took place in a rolling process over the 9 weeks in the camp. The author was present in the camp for the research activities throughout the study. Recruitment of participants for the survey included selecting a tent row at random and visiting each tent in a line from the first tent to the last of rows of tents, approaching groups of women, such as those at water collection points, asking the women who had been approached if they had a sister or neighbor who might be interested to participate, and approaching women who had participated in activities at the primary host agency previously. Study participants were recruited from all six sections of the camp (A–F), with an estimated 700 tents approached out of the 1,549 plots occupied at the time of research (102). Recruitment for the survey was led by a woman from the camp who was an IDP, had worked for the primary host agency, and was trained in how to engage sensitively and confidentially with potentially vulnerable persons.

The 50 interviews and 22 focus group participants were selected from the cohort of survey participants, using both random and purposive sampling. Random sampling involved random selection of approximately 25% of the interview participants from the list of survey respondents. Purposive sampling was engaged with the remaining interview and focus group discussion (FGD) participants to examine participants’ perspectives underlying survey responses, covering each thematic area of the study. Sampling was capped once data saturation had been reached.

A total of 14 interviews were also conducted with 19 key informants including local and international humanitarian responders (8), mental health and psychosocial providers (6), local religious leaders/healers (3), and IDP women that are informal leaders (2) (see **Table 1**). Key informants were selected who could provide insights into the context and the diverse support structures in the women’s socio-ecological environment and who could triangulate findings emerging from surveys and focus groups.

**Table 1:** Key informant interview participants.

In camp		Erbil-based/regional focus	
NGO Camp Management Representative	KII 1	NGO Women’s Programme Manager	KII 2
IDP and NGO Worker Female	KII 4	NGO IDPs Programme Coordinator	KII 3
IDP and Informal Women’s Leader	KII 5	NGO Psychotherapist	KII 6
Primary Imam	KII 9	UN MHPSS Coordinator	KII 7
Supporting Imam	KII 10	UN MHPSS Specialist	KII 8
INGO MHPSS Provider	KII 11	INGO Faith-Based Country Director	KII 14
INGO MHPSS Coordinator	KII 12	NGO Human Rights Branch Manager	KII 17
Traditional Faith Healer Female	KII 13	NGO Human Rights Lawyer	KII 18
UN MHPSS Provider	KII 15		
NGO Protection Camp Focal Point	KII 16		
NGO Camp Management Representative	KII 19		

### 3.2 Data collection

The study included 160 surveys, 50 semi-structured interviews, four focus groups, and 19 key informant interviews. A comprehensive mixed methods approach was selected to enable gathering of empirical data with tools validated among comparable populations while seeking to ensure that the meanings attributed and interpretations made are grounded in the culture, context, and perspectives of study participants, as expressed in their own words (12, 21). The questionnaire (see **Appendix A**) included 69 primary questions, with another 12 follow-up and open-ended questions dependent on the responses to the primary questions. The primary questions included selections from three validated tools with strong psychometric properties. The questionnaire included 14 items from the Harvard Trauma Questionnaire (HTQ)—Iraqi Version (107), the Kessler 10 Psychological Distress Scale (108), and three of the subscales of the Psychological Measure of Islamic Religiousness (PMIR) [the Islamic Positive Religious Coping (IPRC), Punishing Allah Reappraisal (PAR), and Islamic Duty subscales] (109), along with researcher-generated questions^4^.

The questionnaire was organized into 10 domains: Demographics, Conflict experiences (from the HTQ), Distress levels (Kessler-10), self-assessed *Diyana* now and prior to the conflict, Perceived support from others, Beliefs regarding God’s care and proximity, Perceptions of *jinn* (evil spirits) as a cause of suffering, and the Islamic Positive Religious Coping, Islamic Duty, and Punishing Allah Reappraisal subscales (from the PMIR). The way in which the research questions link to the domains of the questionnaire and the tool selection is shown in **Figure 1**. The diagram also indicates which sections of the questionnaire link to the results reported in this paper.

The surveys were conducted one-to-one in Arabic, led by four native Arabic speaking, female Iraqi research assistants who are Sunni Muslims. The research assistants, who were compensated at a daily rate guided by one of the host organizations, are University graduates, recruited through a competitive application process, which was supported by the human resources department of the host. The research assistants’ roles included conducting the survey and data entry, with one of the research assistants likewise serving as the interpreter for an Arabic-to-English transcriber of the semi-structured interviews. Each survey took between 45 and 60 min.

Prior to the start of data collection, the survey tool was translated from English to Arabic and was then reviewed systematically with the research assistants to clarify word meanings and review the appropriateness of terms for the context and population. The tool was jointly reviewed again after piloting with 15 respondents, with alterations made (e.g., adding “witnessed dead bodies” from the HTQ to the survey due to the frequency of this being reported in open-ended portions of the pilot version).

The study adopted a “partial” explanatory sequential mixed methods approach, with the semi-structured interviews and focus-group discussions conducted with participants who had completed the survey and with the responses from those surveys directly informing the focus and content of the interviews and FGDs. Due to time constraints driven by the security context that required monthly renewal of three levels of external written authorizations (e.g., to work in Iraq, pass checkpoints, and work in the camp), the maximum duration of the study was restricted. As a result, the qualitative data collection commenced as a parallel activity following the initial survey piloting period of one week. From that point, three of the research assistants continued surveying, while the author, supported by one of the research assistants who served as the interpreter, conducted all the interviews and focus groups^5^.

Semi-structured interviews were conducted with a sampling of participants who had completed the survey, with questions adapted for each interview using an initial semi-structured interview guide (**Appendix B**) and daily analysis of survey data gathered, to ensure that each of the themes of the study were explored in interviews to the point of data saturation. The relatively high number of interviews (50) were required to achieve data saturation, as 14 of the interviewees were selected in part as a duty of care, where referrals related to suicidality, reports of intimate partner violence, or other protection concerns were required following the review of survey responses. The higher number of interviews ensured that the majority of interviewee participants (36) were not selected in relation to particular vulnerabilities but reflected the diversity of the wider female population in the camp. The questions selected for focus group discussions and key informant interviews (KIIs) were also varied for each session, with questions targeted to serve as explanatory and triangulation tools, examining themes that were emerging in surveys and interviews.

Participants provided informed consent before data collection commenced (**Appendix C**). Surveys and focus groups took place within the gated, host agency compound in the camp, with interviews conducted in a private space within the compound (either within the pre-fabricated office container or in the standalone, enclosed canvas tent). FGDs varied in size from 4 to 10 participants and lasted between 45 and 60 min. Interview lengths varied from 30 to 60 min and were conducted in English and Arabic with the support of two native Arabic speaking, female Iraqi interpreters with backgrounds in the health sector. Interviews were audio-recorded and were subsequently transcribed and translated to English simultaneously by the interpreters that participated in the original interviews.

The research assistants and interpreter received 4 days of training in research ethics, data collection, database use, and concepts related to the study (KR). Each member of the research team also reviewed and signed a Code of Conduct (see **Appendix D**), agreeing to shared values for the research engagement, along with commitments to defined standards of personal conduct, professional integrity, and ethical research.

### 3.3 Data analysis

A database was developed in Microsoft Excel for data entry. Data entry was done daily, with data cleaned in the evenings and at the end of each week. Inductive post-field data analysis was conducted using IBM’s Statistical Package for Social Sciences (SPSS) version 23 (110) for the quantitative data and NVivo 12 for qualitative data (111). Cross-tabulation analysis of survey data was compiled into an Excel worksheet, while results from statistical analysis of relationships between variables within nine domains of the questionnaire and Kessler 10 psychological distress scores, as the dependent variable, were recorded in a summary spreadsheet with supporting tabs. Further sub-group analysis was then conducted within domains to assess whether statistically significant correlations exist between certain response sets and other variables in the study (e.g., having had certain conflict experiences).

Varying cutoff points are suggested for assessing the presence of psychological disorder using the Kessler-10 scale, and many researchers recommend that the Kessler cutoff scores should be adapted to the cultural context (112–115). The K10 uses a Likert scale 1–5 response set to capture how frequently the respondent has felt things such as tired for no good reason, hopeless, so restless/fidgety they could not sit still, and so sad nothing could cheer them up. Once totaled, the scores range from 10 to 50, with 10 being the lowest possible distress score and 50 indicating the most severe distress rating. In this study, the scoring bandwidths suggested by Kessler et al. (112) were used to interpret whether the respondents reported distress levels consistent with a diagnosis of an anxiety disorder and/or depression. These included the following: likely to be well (10–19), likely to have a mild disorder (20–24), likely to have a moderate disorder (25–29), and likely to have a severe disorder (30–50). The cutoff point for the highest level, 30 and above, aligns with a widely used K10 version that has been translated into Arabic, verified by the Transcultural Mental Health Centre in NSW, Australia, and is being used by Australian health departments among diverse populations, including refugees from the Middle East (113).

The primary statistical tests used for quantitative analysis included those focused on determining whether there were statistically significant relationships (or correlations) between two variables (e.g., Pearson’s correlation coefficient or Spearman’s rank correlation coefficient) or statistically significant differences between the average responses of two or more groups (e.g., two independent sample t-test or Wilcoxon–Mann Whitney for two groups and one-way ANOVA test or a Kruskal–Wallis for three or more groups).

In the analysis of the qualitative data, the responses were grouped around nine key research themes: Conflict-Related Stories and Disclosures, Daily Stressors and Impact, Diyana in Life—Proximity, Role, Trajectory, Humanitarian Response, and Responsibilities in Camp, Reason(s) for Suffering, Religious Support—Materials, Spaces and Participation, Religious Support Structures for Women, Coping Actions and Religious Practices, and Vulnerability Factors. Sub-themes or codes were then created to represent the participants’ response themes, with the number and label of sub-themes driven by the breadth and content of the responses.

In this paper, analysis relevant to two of five research questions from the study is reflected (see *Section 1*), drawing primarily (though not exclusively) upon data semi-structured interviews. Other data, pertinent FGD and KII findings, are integrated into analysis on separate research questions (see **Figure 1**) and will be reported in future publications.

### 3.4 Ethics processes and approval

The project secured ethical approval for research with human subjects through the University Research Ethics Committee (UREC) of Queen Margaret University in Musselburgh, Scotland, and was implemented in compliance with UK and global research ethics standards. At the time of the study, there were no formalized national ethics approval processes within Iraq (116, 117). Given the distressing context of life in the camp and the potentially sensitive nature of the research topics, steps were taken to minimize the potential for retraumatization of participants and to ensure the comfort of the participant throughout the research. Participants were not asked to recount details of conflict or displacement experiences (although many prioritized sharing these narratives and were given space to do so), and the research team did not refer directly to ISIS (or *Daesh*, in local terminology), instead referring to “the events” if the 2014–2017 period was referenced. Additionally, verbal consent (rather than written) was sought to prevent undue alarm in being asked to sign a formal paper in a context where most of the women could not read the paper (due to low literacy), where many women perceived that they or their family members were under suspicion and threat of arrest as former ISIS affiliates due to being residents of Mosul during the occupation and in a context where gender norms sometimes require that male heads of household be the primary contact point for interactions with non-family members.

Throughout the study, the wellbeing of participants was prioritized, with the general affect of individuals, as seen in their body language, facial expressions, and tone of voice, closely observed and data collection paused or stopped if participants became upset or ill at ease. All research participants received referral information to enable access to post-research emotional support, if desired, from a trained staff member of the host agency. A total of 10 participants were referred, with their consent, to specialist mental health care agencies in the camp, following references to suicidal thoughts or attempts, or expressing acute psychological distress, during surveys or interviews. Five additional protection-related referrals were facilitated. All names of participants used in publications have been changed, with pseudonyms ascribed.

## 4 Results

### 4.1 Participants

#### 4.1.1 Demographics

A total of 160 women living in the IDP camp participated in the study. All 160 participants were surveyed, with 50 of the initial cohort participating in subsequent interviews and 22 of the cohort also participating in focus group discussions. Participants were between the ages of 18 and 64 years old, with an average age of 34 (see **Table 2**). All of the women were Iraqi nationals and identified as practicing Sunni Muslims. A total of 92 women (58% of the total cohort) were married with their husbands in a known location (either in the camp, in a nearby town or in jail), 41 women (26%) were widows, 13 women (8%) were married with husbands missing, 11 women (7%) had never been married, and three women (2%) reported being divorced (see **Table 2**). A total of 75 women (47%) were head of their household in the camp. The average number of children per household was 4 (see **Table 2**). A total of 19 faith leaders, MHPSS, and humanitarian workers were also interviewed as key informants (see **Table 1**).

**Table 2:** Summary of participant socio-demographics.

Variable	No.	%
Gender		
Women	160	100%
Nationality		
Iraqi	160	100%
Religion		
Sunni Islam	160	100%
Age*		
18–19	6	4%
20–29	54	34%
30–39	57	36%
40–49	30	19%
50–59	12	8%
60–64	1	1%
Relational status		
Married	92	58%
Widow	41	26%
Husband missing	13	8%
Divorced	3	2%
Never married	11	7%
Head of household		
Yes	75	47%
No	85	53%
# of children per household		
0	8	5%
1	16	10%
2	15	9%
3	27	17%
4	25	16%
5	20	13%
6	18	11%
7	19	12%
8	6	4%
More than 8	4	3%
Missing data	2	1%

*Includes allocation of 18 who did not know exact age to the decade of estimated age.

#### 4.1.2 Conflict experiences

The conflict events experienced by the women, based on the 14 items from the Harvard Trauma Questionnaire (HTQ) utilized in the survey, are detailed in **Table 3**. An analysis of the mean number of conflict events experienced by each participant in the cohort found that participants had experienced an average of 8 of the 14 conflict events listed.

**Table 3:** Duration and frequency of displacement.

Majority displaced when	
Initial ISIS Emergence in 2014	22%
Liberation Period 2016–2017	70%
Other	8%

# of times displaced	
More than 5 times	11%
Two or more times	78%

Length of time in camp	
Average # of months in camp	19
In camp more than 1 year	75%
In camp more than 2 years	32%

#### 4.1.3 Displacement experiences

All of the women had been displaced as a result of the ISIS conflict; 92% of the women had been displaced for more than a year; 22% had been displaced since 2014 when ISIS emerged in Iraq, and 70% had been displaced since the period of the battle to liberate Mosul between 2016 and 2017; and 8% had displaced in the years following the liberation of Mosul (see **Table 4**). A total of 89% of the women had experienced secondary displacement, meaning that they had been displaced more than once following their initial displacement from their home locations (see **Table 4**). The average duration of time the women had resided in the IDP camp where the study was conducted was 19 months (see **Table 4**).

**Table 4:** Conflict events experienced.

By category	
Religious	Yes
Oppressed because not religious enough*	28%
Witnessed destruction of religious shrines or places of religious instruction**	44%
Basic needs deficits	
Suffered poor health without access to medicine or healthcare	76%
Suffered from lack of food or clean water	91%
Lacked shelter	71%
Personal losses	
Property stolen, confiscated, or destroyed	89%
Death of a family member	39%
Death of a friend	56%
Disappearance of a family member (child, spouse, etc.)	29%
Combat experiences	
Confined to home because of chaos and violence outside	90%
Exposed to combat situation (gunfire, explosions, artillery fire, shelling) or landmines	88%
Experienced serious physical injury	5%
Serious physical injury of family member or friend	42%
Witnessed dead bodies or human remains^	59%

*Four participants prefer not to answer/skipped.

**One participant could not remember.

^Calculations based on 153 surveys as question was added after pilot.

In the interviews, daily stressors (118) were frequently referenced by participants as primary causes of current distress. The harshness of daily existence and the depth of fatigue, physical deprivation, and feelings of hopelessness that resulted from daily life in the camp (see *Context*) were frequently the focus when reflections of anguish and questioning about God’s care were voiced (see *Section 4.2.2*). The breadth of material, social, and psychological stressors referenced in the interviews are shown in **Tables 5**–**7**, with the frequency of interview participants mentioning the stressors noted. The role of daily stressors as a primary driver of poor wellbeing according to the study participants, along with the function of “spiritual stressors” and faith as a means of coping with daily stressors, will be reported in future publications.

**Table 5:** Material stressors described as adversely impacting wellbeing.

Material stressors	# of participants referencing
Blocked from returning home	
Blocked from returning home—home destroyed, high rent, limited income in places of origin	9
Blocked from returning home—due to hostile neighbors and/or insecurity in place of origin	3
Blocked from returning home—due to government prohibition, houses in disputed territory	4
Blocked from returning home—home confiscated due to familial ISIS Affiliation	1
Inadequate services and/or living conditions in the camp	
Recurring/persistent illness within family—medicines and/or surgeries inaccessible	8
Limited income opportunities in camp	6
High household debt levels	5
Hunger—insufficient food	5
Widow-focused policies in aid targeting—those missing husbands and older widows neglected	4
Extreme cold in winter, extreme heat in summer—difficulty keeping warm/cool	4
Lack of electricity	3
Maltreatment from police managing gate and/or camp staff overseeing distributions	3
Missing identification papers	3
Tent problems (i.e., in poor repair, unable to afford poles to increase tent height/mobility within)	2
Cost of goods in camp high	2
Every day the same	2
Loss of access to social security payments and pensions (due to loss of documents, spouse, etc.)	1

**Table 6:** Social stressors described as adversely impacting wellbeing.

Social stressors	# of participants referencing
Forced, extended separation from family due to displacement	13
Children wanting things day to day that the women, as parents, cannot provide	12
Family abandonment—by family outside of the camp	7
Difficulties with male family member (conflict—intimate partner violence)	6
Family rejection—by other family inside of camp	5
Pressure by family outside camp to abandon children to other family and leave camp	4
Children misbehaving, not doing well	3
Family outside of camp too poor or with ill health and unable to support	3

**Table 7:** Psychological stressors described as adversely impacting wellbeing.

Psychological stressors	# of participants referencing
Worry about a family member still missing	9
Worry about a family member in prison	7
Worry about future—will it ever get better, what will we do if evicted	6
Sadness over loss of dream or career	4

#### 4.1.4 Distress levels—Kessler 10 and local idioms of distress

The Kessler 10 (K10) Psychological Distress Scale (108) and two local idioms for distress—Tired Soul (*Nafseetak ta’bana*) and Heart Squeezed (*Qalbak Maqbood*) from the Harvard Trauma Questionnaire, Iraqi version (107)—were used to assess participant’s perception of their health and wellbeing in the 4 weeks prior to the study. “Tired soul” is a local expression reflecting a lived experience comparable to what is described as moderate to severe depression in clinical terminology, while “heart squeezed” describes a lived experience related to anxiety, worry, and anguish.

**Figure 2:**
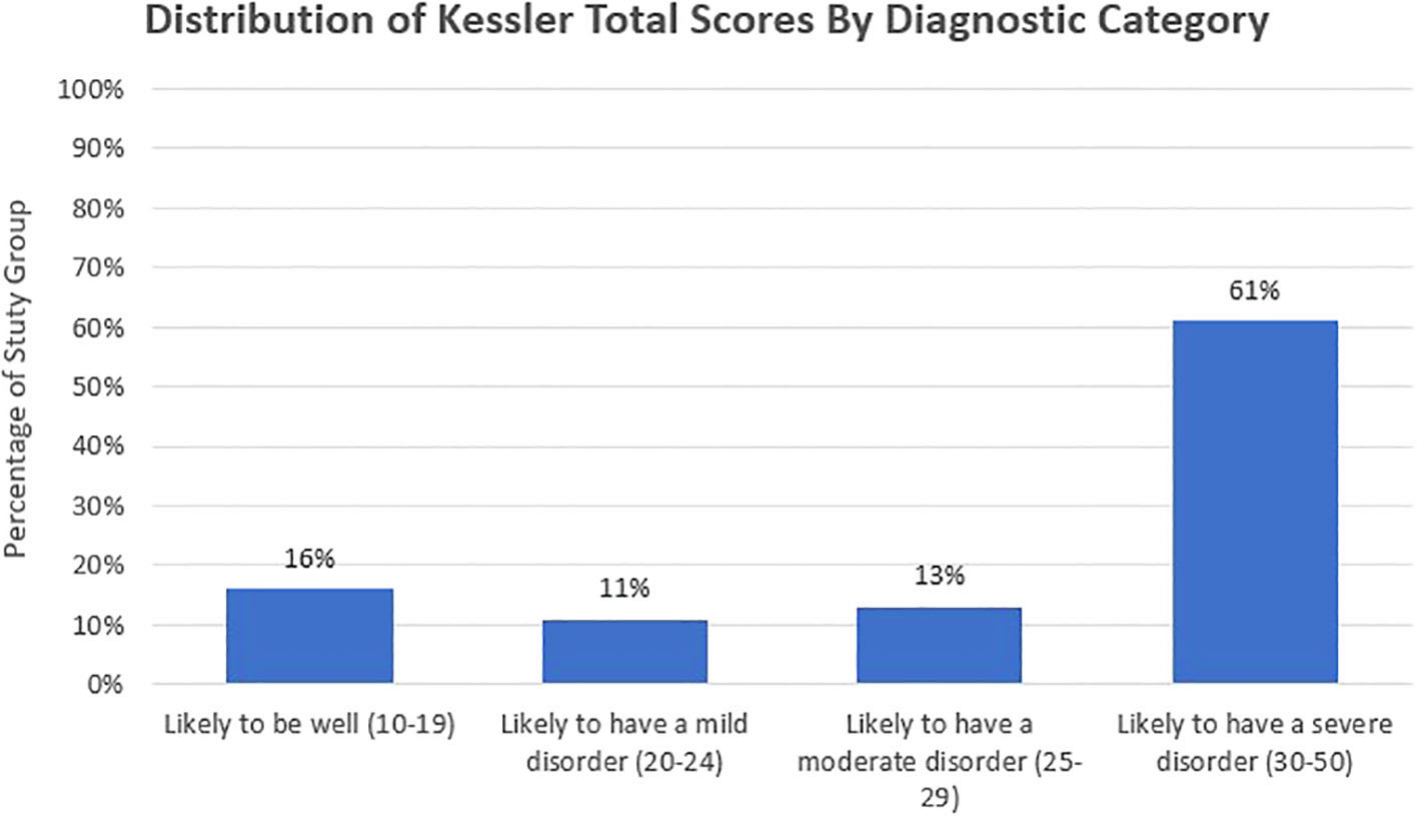
The distribution of Kessler-10 psychological distress scores, grouped according to diagnostic cut-off points, showed that 118 women (74%) in the study reported distress levels that indicate they are likely to have a moderate or severe anxiety disorder or depression.

The average Kessler score for the study group was 31.43, within the highest scoring bandwidth, indicating very high distress among the women and symptomology aligned with severe anxiety disorder(s) and/or severe depression. The distribution of responses shows that 118 women (74%) in the study report distress levels in the study reported distress levels that indicate that they are likely to have a moderate or severe anxiety disorder or depression (see **Figure 2**).

**Figure 3:**
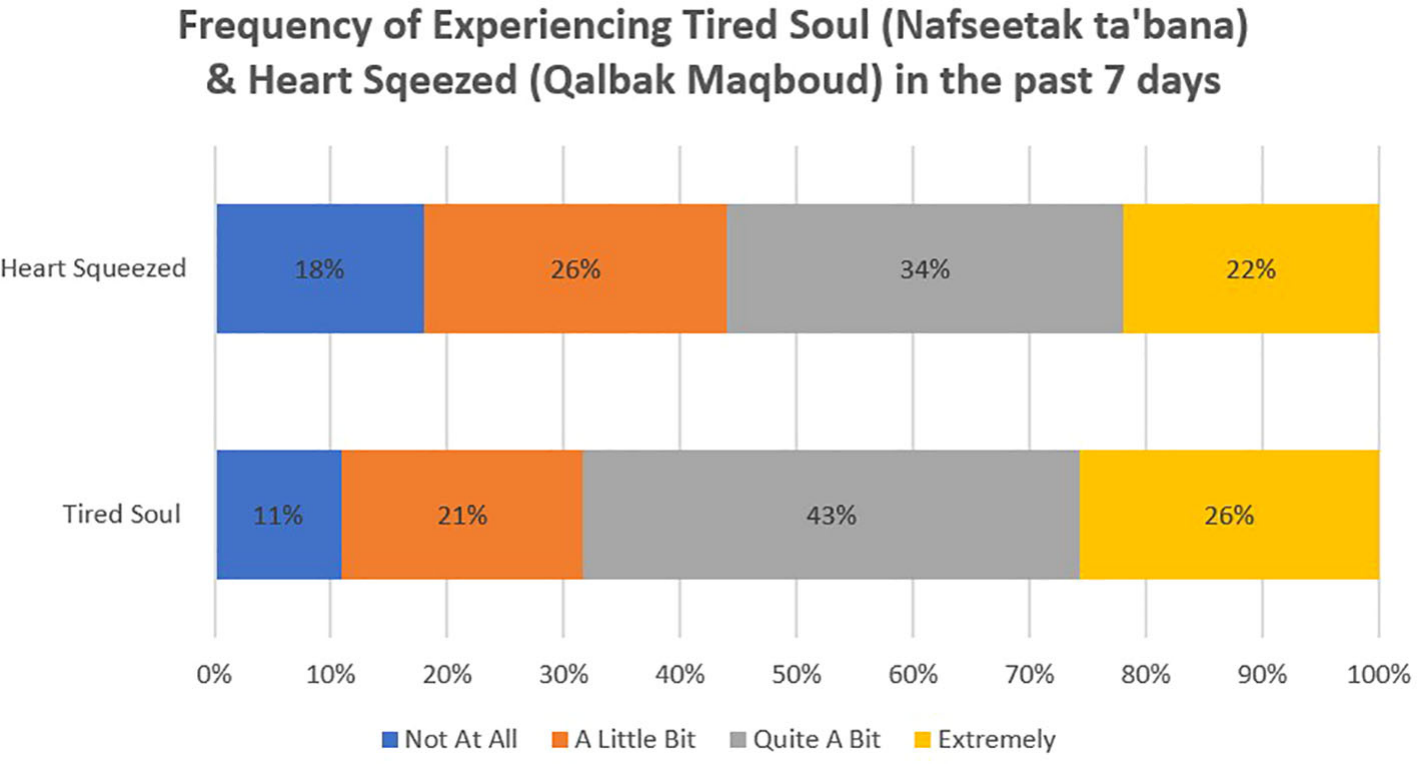
The distribution of responses regarding distress levels using local idioms showed that 56% of the women felt quite a bit or extremely “bothered” by feeling their “heart squeezed” (a local expression for experiences similar to anxiety) and 68% felt quite a bit or extremely “bothered” by having “tired soul” (a phrase that describes experiences similar to depression).

Responses to distress level questions using local idioms for distress mirrored the elevated levels demonstrated in the total Kessler scores (see **Figure 3**). A total of 109 women (68%) reported being “Quite a Bit” or “Extremely” bothered by Tired Soul (*Nafseetak ta’bana*), while 90 women (56%) reported being “Quite a Bit” or “Extremely” bothered by Heart Squeezed (*Qalbak Maqbood*). Of the women, 19% (n=31) responded that they were “Extremely” bothered by both Heart Squeezed and Tired Soul, and 9% (n 14) said that they did not feel bothered by either “at all”.

### 4.2 Self-appraised faith levels and beliefs regarding “God’s care” linked to wellbeing

To assess the relationship (if any) between the religious meaning system and practices of the women in the study and their reported levels of distress and perceptions of wellbeing, it was important first to understand how “faith” was defined by the women, the degree of value attributed to it (if any), and their own self-perceptions regarding the level of their faith. It was in fact the perceptions of their own faith level at the time of the study, the meanings attributed to the role of faith practice in relation to attaining Paradise and “beliefs in God’s care” that were strongly associated to distress levels and wellbeing. The following sections expound upon the findings relevant to these themes.

#### 4.2.1 Current faith levels, definitions of faith, and distress associations

**Figure 4:**
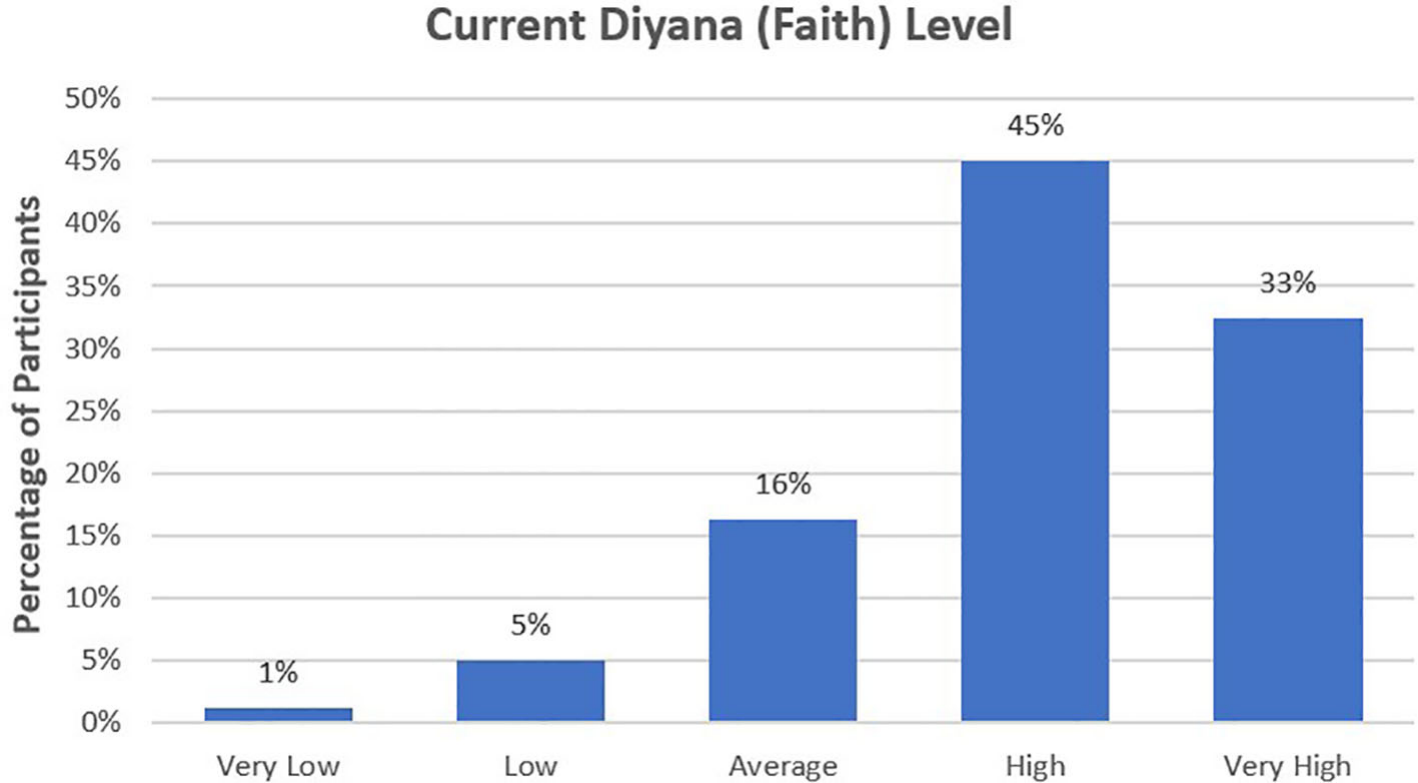
Self-perceptions of faith levels showed a high level of religiosity, with 78% describing their faith—or *diyana*, using local terminology—as high or very high at the time of the study.

Of the women in the study, 78% assessed their faith, or *diyana*, as high or very high at the time of the survey. The remaining respondents appraised their *diyana* at the time of the study as average (16%; n=26), low (5%; n=8), or very low (1%; n=2) (see **Figure 4**).

*Diyana* is the local term for faith that represents several factors: one’s internal orientation toward religion, one’s beliefs, one’s sense of closeness with Allah, and one’s religious practices. The word is derived from ancient Mesopotamian languages, particularly regional Assyrian language dialects, and its use in modern day appears to be localized to parts of Iraq (119). The meaning of the term is rooted in words referring to judge, judgment, and particularly Allah as judge (119). The descriptions of faith shared by the women reflect the linguistic roots of *diyana*, as faith levels were interpreted through a framework where one will be judged after death for actions taking in this life, particularly how well one lived in accordance with Islamic teaching.

The study participants’ personal descriptions of what high and very high *diyana* entailed included the fulfilment of most or all of the obligatory practices [with an emphasis on fulfilling the five daily prayers (*salat*) and fasting during the month of Ramadan]. Undertaking additional Sunnah prayers and fasts, such as fasting Monday and Thursday, on the White Days of each month (the 3 days in the middle of the Arabic month, when the moon is a full moon), and 6 days during *Sjawal* (the month after Ramadan) (46) despite experiencing persistent hunger, distress, and logistical constraints in the camp, were seen as an indicator of very high *diyana*.

Some women expressed that they had average or low *diyana*, for example, because they were participating in behaviors that they perceived to be either forbidden (*haram*) or discouraged for devout believers, behaviors such as “gossiping,” wearing “modern” clothes instead of hijabs, raising their voice at their children, and watching television or listening to music that they felt they should not be, while others talked about their Qur’an reading as less now and judged their *diyana* to be less accordingly.

**Figure 5:**
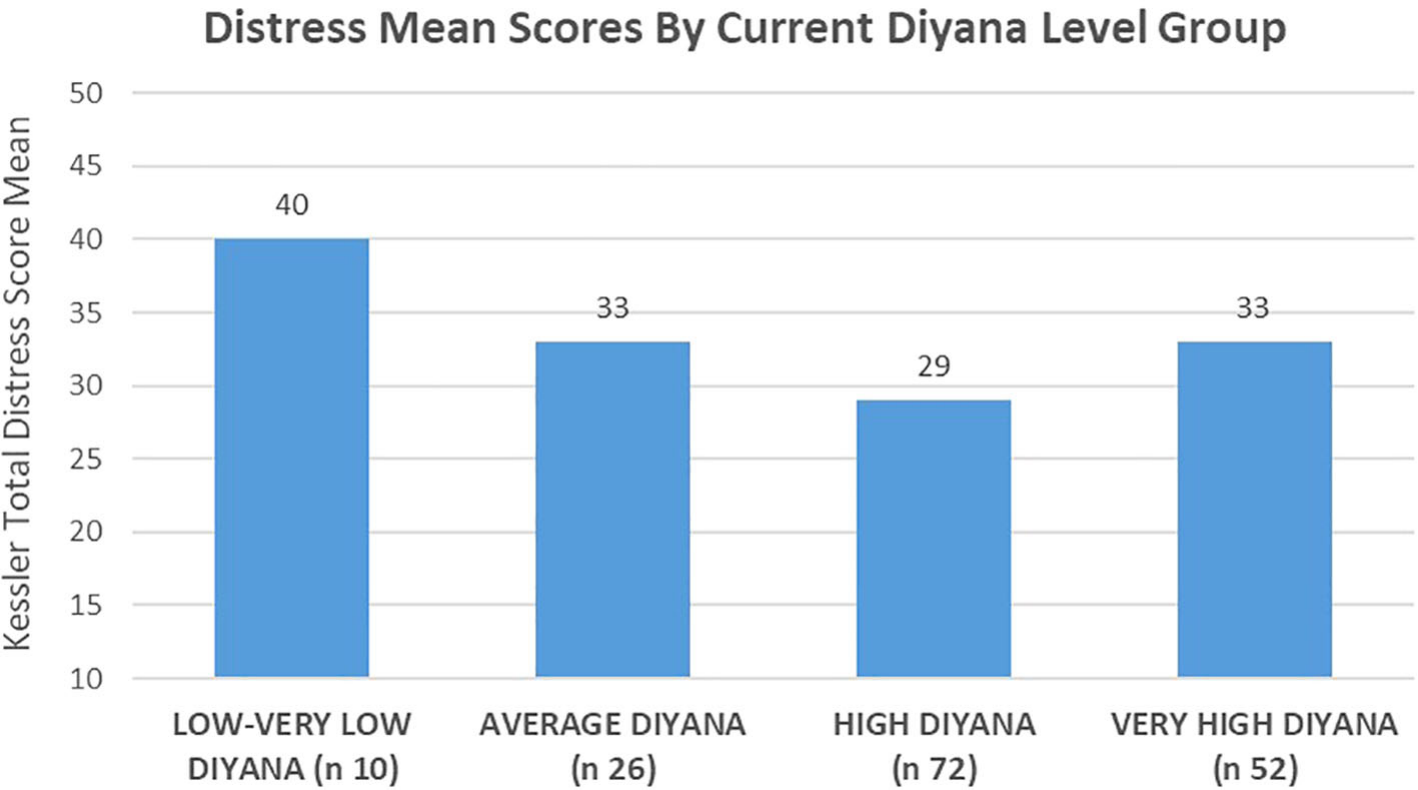
Self-appraised current “faith-levels” were associated with Kessler psychological distress score averages to a highly significant degree, with the 10 women who perceived their *diyana* to be “low or very low” scoring the highest among the comparison groups.

Self-appraisals of “current” faith levels were associated with K-10 distress scores to a highly significant degree. The 10 women in the study that felt that their *diyana* was “low or very low” had the highest distress scores of the three groups, with a mean score of 40 (see **Figure 5**). This score is nearly 9 points higher than the distress mean (31.43) for the wider study population (one-way ANOVA/F=5.562/*p*=.001). The results of the relationship between high and very high faith perceptions showed that women who assessed their faith as high had the lowest Kessler distress mean at 29, while women who felt their *diyana* was very high in fact had slightly higher distress scores (mean = 33).

While the associations between distress scores and perceptions of faith are statistically significant, the “direction” of that relationship, in terms of causality, requires additional enquiry. When taking the qualitative data into account, the finding may indicate that as distress increases, the fervency of faith practice increases among persons who are devout. The finding may also point to the role that underlying existential anxiety may play in elevating faith practice. Many in the study expressed a belief that adverse events (such as displacement) during life may be a punishment for poor *diyana* and that their ultimate destiny after death in regard to attaining Paradise with God was contingent upon fulfilling the required Islamic duties. Further research is needed to clarify these initial reflections, however.

#### 4.2.2 Mental health impacts of faith practice linked to perceptions of God’s care

In addition to examining the relationship between perceptions of faith and reported distress and wellbeing, this study explored which types of religious practices are linked with higher and lower levels of distress. In response to the Islamic Duty subscale, which assessed the frequency of fulfilling required religious practices, along with voluntary “*Sunnah*” actions (see *Section 1*), there was a high frequency of religious practice reported (see **Table 8**).

**Table 8:** Religious practices frequency—Islamic duty subscale.

How often do you pray		
Never	0	0%
Few times a year	3	2%
Several times a month	2	1%
Several times a week	9	6%
Most of the time the five daily prayers	41	26%
Five times a day or more	105	66%

How often do you fast		
Never	1	1%
A few times in life	5	3%
Few days of the month of Ramadan each year	1	1%
Half to all the month of Ramadan each year	3	2%
The whole month of Ramadan each year	120	75%
Other religious days or sunnah fasts in	30	19%
in addition to Ramadan		

Except in prayers, how often do you read or listen to the Holy Qur’an		
Never	4	3%
A few times in life	2	1%
A few times a year	2	1%
A few times a month	14	9%
About once or twice a week	44	28%
Once a day or more	94	59%

Except in prayers, how often *d’iker* or *tasbih*		
Never	7	4%
A few times in life	1	1%
A few times a year	2	1%
A few times a month	7	4%
About once or twice a week	14	9%
Once a day or more	129	81%

The Islamic Duty subscale also includes a question regarding frequency of attendance at the *masjid* (mosque). No prayer space had been made available for women in the camp; thus, this question was removed. In response to a separate question as to whether they would attend a prayer space if one was available for women 65% said yes and that having such a space would be beneficial for coping.

Statistical analysis of the total scores from the Islamic Duty subscale and the total Kessler-10 scores did not indicate a statistically significant association. However, subsequent analysis at the response-group level showed a statistically significant link between frequency of prayer and the Kessler question regarding the feelings of hopelessness [χ^2^(4) = 10.058, *p*=.039]. Pairwise comparisons, using Dunn’s (1964) procedure with a Bonferroni correction for multiple comparisons, showed that those who prayed “Most of the time the five daily prayers” had a lower mean rank for how often they had felt hopelessness in the 4 weeks before the survey (67.30) compared to those who prayed “Several times a week” (115.33; *p*=.039) (120, 121).

Qualitative findings likewise illustrated that faith-practices, such as prayer, fasting, reading, and reciting the Qur’an and charitable giving, were highly prized by the study group, in part because they were seen to be conduits of exchange with the ultimate Provider and Protector, a relationship through which comfort, guidance, divine protection, and provision for basic needs could be secured.

Shorash, a 37-year-old widow and mother of four children, said she recites *Ayat Al-Kursi* (the Throne Verse Qur’an 2:225) to invoke divine protection over her children, for example, while 23-year-old Shaima said she does *tasbih* (repetitive prayer rituals, using prayers beads or counting on fingers) to help alleviate the physical sensation of her rapid heartbeat when she has increased anxiety. Other women, like Hanan, a widow in her 40s, pray for provision for daily needs for food, funds, or for physical healing when they did not have medicine. “Everyone who fasts, prays, reads the Qur’an and makes *dua* (spontaneous utterances glorifying God) is close to Allah,” Hanan explained.

Across the study group, qualitative data show that the degree to which faith practices facilitated distress alleviation was associated, as Hanan indicated, to the degree to which the practices facilitated a sense of nearness to Allah through which the participants felt they received those benefits.

Those who pursued non-obligatory practices, or “extra” duties, such as Sunnah fasts and prayers, frequently shared perceptions of greater fulfillment and comfort derived. This was the case for 30-year-old Rasha, who said she does “all of the mandatory practices” along with “extra things”.

“If you pray you will stand in front of God and no one hears you or sees you just God and that makes you feel close and feel better,” the mother of five children explained. Her family had been displaced for more than five years.

In the context of deprivation and hardship in the camp, for those who felt a sense of “nearness” to God combined with the perception that they were living as they ought to live—fulfilling the five pillars of Islam, undertaking voluntary devotional activities, thanking Allah when faced with adversities, and avoiding forbidden or undesirable behaviors in day to day life—faith practice was felt to be a lifeline for coping and survival. As 51-year-old Jinan explained:

Without the patience that we get from Allah we will be mad. Praise God, I pray, read Qur’an and fast each Monday and Thursday. It’s just like pouring water on fire. My heart becomes very comfortable. I pray, make *dua* (spontaneous utterances glorifying God) and cry and after I feel much better … I don’t have anyone else but I have God. Without God, I can’t face it and stay alive.—Jinan, 51 years, three sons killed in Mosul bombings.

Conversely, for those who did not feel that nearness despite a high frequency of faith practice or felt that the frequency of their practice was insufficient, these reflections were powerful sources of distress and pain, with the subsequent anxiety regarding the threat to entering Paradise on the Day of Judgment because of the dissonance, adding oppressiveness to the grief. “Because [I don’t pray], it affects me directly,” explained Khalida, a 35-year-old widow and mother of three. “I feel the whole time that I have something heavy on my chest—and I feel I can’t breathe”.

For 30-year-old Hosa, and other women in the study who expressed feeling a loss of closeness or that God seems unresponsive to prayer, this perceived loss of connectedness with God was consistently identified as a primary source of psychological distress. “Why before the events I felt God near me and now when I need him, I do not feel this? It adds sadness on my sorrow,” she said, crying heavily in the interview. Before the conflict, she said she had often done “extra” prayers and read the Qur’an frequently, but after being displaced, the death of family members, and the arrest of her husband, she said that for a time, she started to lose her sense of closeness to God and her religious practice reduced. She is alarmed now that, despite resuming her faith practices, she has not regained the sense of nearness to God. When asked if she thinks her concerns about *diyana* are linked in any way to the “depression” she is feeling, she said: “It’s the center”.

**Figure 6:**
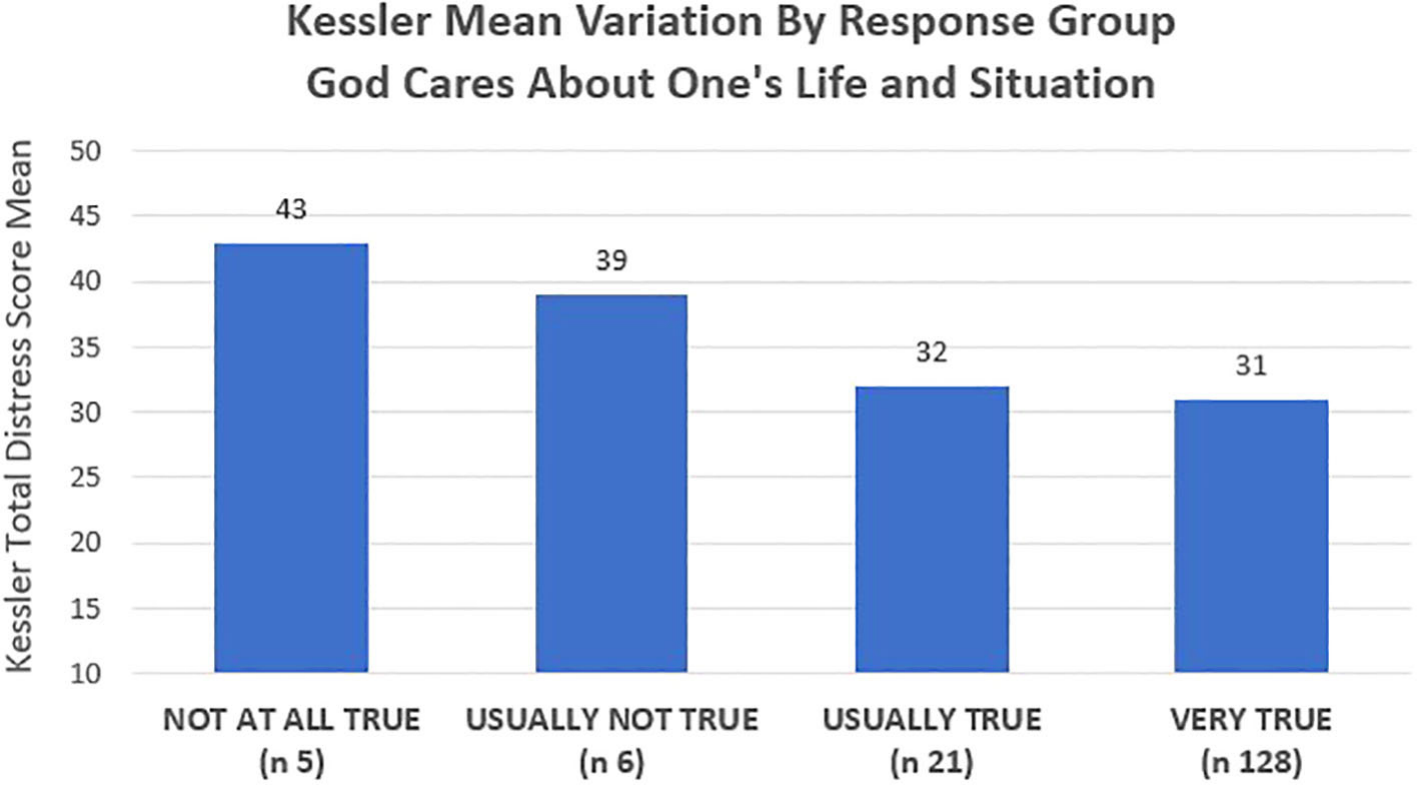
Statistical analysis comparing the respondents average distress scores and the degree to which they perceived that “God cares about my life and situation”, showed that those that believed the statement was “not at all true” had the highest distress mean scores of any group assessed in the entirety of the study.

In addition to the qualitative evidence pointing to perceptions of nearness and responsiveness of Allah as a key factor influencing the function and impact of faith practices, statistical analysis likewise showed that beliefs regarding “God’s care” for one’s life and situation were measurably associated with Kessler distress scores (see **Figure 6**).

Women who responded “Very true” (n = 128) to the statement “God cares about my life and situation” had the lowest distress mean score (= 31) in comparison to the other response groups. Those who reported that it was “Usually true” (n = 21) had the second lowest distress mean (score = 32), with the distress means of those who felt it was “Usually not true” (n = 6) scoring to 39 on the Kessler scale. Those who said it was “Not at all true” (n = 5) had the highest distress scores of any response group assessed in the study, with a mean Kessler distress score of 43 (one-way ANOVA/F = 3.735/*p* = .013).

#### 4.2.3 Gender barriers to fulfilling faith practices deepened distress

The function of faith practice was found to be influenced by gender-related access constraints in the camp gender. Despite the high prioritization and valuing of faith-related practices to aid coping with the extreme distress of life in the camp among women in the camp, poor gender sensitivity and poor religious literacy among some camp managers, generalist aid workers, MHPSS providers, and the local faith leaders in the camp created barriers to faith practice that compounded feelings of distress in some women. Throughout the study, many women shared a desire for a specific range of spiritual supports that were expressed as essential to ensuring life in camp along with the losses suffered during the conflict itself. The supports prioritized included a prayer space for women in the camp (desired by 65% of the study respondents; there is only a prayer space for men), a gender-segregated public space to gather with other women for social support and to charge phones that are needed to listen to the Qur’an (as many of the women are not literate), professional counseling in which they are able to discuss the concerns about their faith that are causing them distress, copies of the Qur’an (preferably with the electronic reader pen for women with low literacy to use), and space and support to form and strengthen support networks with local women of faith in the camp who can provide consolation and guidance in gender-segregated spaces.

In the two years that the camp has been operating, women had not been directly assessed and asked about their coping priorities (faith or otherwise). While camp management had allowed external religious actors from the region to construct a tented mosque space and to work with the male imam to distribute religious materials, gender-aware approaches would have acknowledged that gender norms prohibit women from making contact with men outside of their families. There are exceptions made at times, however; the prevailing cultural norms means that any aid efforts that are coordinated only with the male formal religious leader are likely to result in exclusion of families headed by women who are heads of household. In this post-conflict context, where many men were killed, missing, or detained, 34% of the households in the camp were headed by women (104). Poor awareness of how gender norms shape access thus resulted in men in the camp receiving greater support for their faith-related needs than women.

According to study respondents, they were “aware” that they should not discuss their anxiety about their faith with MHPSS providers during counseling sessions and aid workers in general in regards to other sectors in the camp, and thus, despite the importance placed on these concerns by many, the women frequently avoid these subjects. When asked how they know they are not “meant” to discuss faith with aid workers, 24-year-old Madiha explained, “it is clear, because when they talk to us they never mention anything about religion … but,” the mother of five continued, “I wish they would talk about religion and practices because it is very important”.

Additionally, study respondents shared that the lack of prayer space, limited access to religious materials, and the lack of public gender-segregated space for women to be able to gather to build social and spiritual support networks compounded feelings of disempowerment, helplessness, and emotional anguish. Tamara, a 39-year-old mother of four, shared that she had attended her local mosque regularly before displacement, as it helped her feel connected to other women and to God, adding “you feel you are nearer to God, more than in the house.” She said that she does not understand why she is not allowed to attend prayer now in the camp. For 29-year-old Hana, not having access to the prayer space was also a source of sadness, but the fact that women in the camp had not been asked about their faith support needs in the previous 2 years deepened her frustration. “I wish I had been asked,” she said, “not only (by) aid workers; the imam of the mosque, he should be responsible for those things”.

## 5 Discussion

This research is among a small segment of studies that provides both qualitative and quantitative evidence regarding the potent influence of religious meaning systems and coping approaches on the mental health and perceived wellbeing of displaced Muslim women affected by conflict. Such quantitative data on these themes among comparable populations within aid contexts, in particular, remain relatively scarce in research literature. This analysis examined the function of faith practices in distress alleviation, through exploring the relationship between the locally defined conceptualization of wellbeing, the religious meanings attributed to faith practice, and the role of secure attachment to Allah (sense of nearness, beliefs in God’s care and responsiveness) in influencing the function and distress-related impacts of faith practice. This was shown in the examination of Kessler 10 Psychological Distress Scores, two local idioms of distress and perceived wellbeing as variables strongly influenced by 1) self-appraised faith levels post-displacement and 2) perceived changes to faith.

### 5.1 The pathway to wellbeing defined by religious meanings

Faith was shown to be a primary framework that shapes the goals of life and through which life was appraised by the women in the study. It is not the only element in that framework, as culture and other factors influence strongly; however, it is potent and pervasive. Religious meanings were attributed to everything from the cause of suffering to illness and the weather. The study participants communicated strongly that faith and specifically Allah, seen to be the creator and director of all things who will judge at the end of life, is a gravitational center of their worldview. This faith, or *diyana*, was multifaceted, incorporating assent to the teachings of Islam, performing religious practices, and an internal orientation of one’s proximity or attachment to Allah. Self-appraisals regarding “strength” of faith referred to the degree of fulfilment of required and voluntary faith practices of Islam (the five pillars and Sunnah, respectively) and were described frequently in terms of a sense of closeness (or distance) and in active terms where Allah “helps,” “listens,” or is felt not to be doing so. None of the 160 women in the study expressed disbelief in Allah or indicated that faith is not relevant.

Other studies indicate that this type of faith integration in thought, feeling, and action, with decisions influenced heavily by the directives of religious law, typifies Islamic faith. Islam is “spread across the whole being,” states Dasti and Sitwat (28, p. 51). “It is a deen (way of life) yielding guidelines for its followers in each domain of life” (28, p. 51). Similarly, Mahmood (43) describes the integration of faith within the Islamic worldview, stating that “Islam is a way of life and Islamic teachings via the Qur’an and Hadith provide guidance for tackling life’s challenges … Health and wellness are view holistically by Muslims” (p. 265).

Comfort and resolve derived from faith by many of the women was found in part by identification with the faith narrative and locating themselves within that unfolding narrative. Many of the women placed themselves within the story of Allah’s plan for the world and for their lives and looked to the life and teachings of the Prophet Mohammed for guidance (including following his examples for fasting and praying dua that the Prophet Mohammed prayed). Mental health providers seeking to care for displaced Muslim women may consider ways to support the individual’s self-identification within the over-arching divine narrative of their faith, with the purpose for human existence inherent within the narrative and with founders and “heroes” of the faith in sacred texts who encountered trials but ultimately prevailed.

This finding reinforces the view of other research among displaced Muslim women and populations of faith that has found that identification of one’s participation within a “master narrative” (122, p. 49) of faith can serve as “grounding principle” (123, p. 565) that can have therapeutic benefit (2, 7, 15). The ability of persons of faith to find their own place within a wider religious narrative and to identify with stories within oral tradition or sacred texts of faith founders, “heroes” of faith, saints, and/or ancestors who experienced adversities and suffering in their lifetimes but ultimately triumphed provides a foundation for positive reappraisal (7, 124–127).

Embedded within that narrative, the deeply rooted belief regarding the Day of Judgment, and the hopes and fears springing from that belief, was among the most profound influencers of decision-making, distress, and perceived wellbeing of the women that were observed in this study. The theme came through strongly that the women’s beliefs regarding the afterlife were highly potent in the influence on the appraisal process and in shaping the locally held understandings of wellbeing and primary goals or global goals of life (27, 128). This “Paradise Orientation,” based on the Islamic teaching that Allah will determine who will be with him forever in Paradise based on the actions individuals took in their lives, was directly associated with the meanings attributed to life events and informed decisions they are making in the present, including which coping strategies (faith and non-faith) to engage or avoid.

Perceptions of wellbeing were thus described by study participants as an internal sense of the pleasure and proximity of Allah, with this internal perception linked to self-appraisals in which individuals felt they were living life according to Allah’s commands as set forth in Islamic teaching. The emphasis placed on both practice and proximity now, however, was given significance because of their perceived role in facilitating the pathway to the ultimate goal of life, being permitted by God to enter Paradise after death, where there will be no more suffering, only complete peace and unbroken communion with Allah for eternity.

Because of the pervasive significance of this “Paradise Orientation” in shaping the study participants’ global goals for life, definitions of wellbeing, coping decisions, and reappraisal processes, all of which directly influenced their distress levels and perceived wellbeing, this concept is suggested by this study as a potential area of focus for mental healthcare providers working with displaced Muslim women. Assessing and seeking to understand the individual’s beliefs regarding the afterlife and the individual’s perception of their position in relation to receiving the reward of Paradise after the Day of Judgment may unearth underlying causes of distress that may be contributing to poor mental health status.

The findings of this study regarding the significant degree to which the conceptualization of wellbeing is shaped by the religious meaning system among devout faith groups reinforce the findings in works by religious scholars, anthropologists, and other academics (38, 39, 43, 45). Similarly, other studies have noted that beliefs regarding the afterlife influence reappraisal and interpretations of suffering (6, 43, 56, 129, 130). Research that documents and clarifies the pervasive impact of the orientation to Paradise as it relates to coping decisions and mental health outcomes is not noted elsewhere in existing religious coping literature.

### 5.2 Beliefs regarding God’s care mediate the distress impact of faith practice

There was a high frequency of religious practice reported by the women in the study. Qualitative data regarding faith practices, distress, and wellbeing underscored that, while distress scores (and corresponding indications of depression and anxiety) remained high among the study group, driven in part by numerous daily stressors (118) in the displacement context, faith practices were perceived to be vital, primary means of coping by many in the study. Participation in the daily prayers (*salat*); supplemental, spontaneous prayers to Allah (such as *dua* and *tasbih*); reading and reciting verses from the Qur’an; and fasting were widely reported to be critical means of soothing anxious feelings, facilitating a sense of nearness to Allah, which in turn brought comfort, and receiving spiritual protection and material provision directly from God. Those who pursued non-obligatory practices, or “extra” duties, such as Sunnah fasts and prayers, shared perceptions of greater fulfillment and comfort derived.

The high level of religiosity observed in the study aligns with the levels observed among Muslims in the Middle East in general (131) and those observed by other studies among Muslim women facing adversity in humanitarian and forced migration contexts (6, 11, 13, 73, 75). Furthermore, the functions described, with religious practices providing comfort, alleviating anxiety, providing a sense of internal control, and enabling the petitioner to access divine power and protection in their lives, align with the resilience promoting functions of faith identified in other studies (6, 13, 73–75, 132, 133).

The extent to which the underlying belief systems regarding divine control and Paradise Orientation, along with the frequency of faith practice, influenced perceived wellbeing and distress levels, was mediated by study participants’ perception of God’s nearness and God’s care for their life and situation. When beliefs functioned as promoters of resilience, they were spoken of most frequently in relational terms, discussed as closeness, being heard, not being alone, and being provided for by God, in response to unique, personal requests for help. For those who spoke of feelings that God is unresponsive and does not care, grief and anguish were frequently expressed, particularly if the individuals had felt near to God at some point in their lives previously. In statistical analysis, as detailed in *Section 4.2.2*, beliefs regarding God’s care for one’s life and situation were associated with Kessler distress scores, with those stating it was “Not at all true” that God cares about their life and situation having mean scores that were 12 points higher than those who felt it was “Very true” that God cares.

Other research has likewise identified that the role of faith beliefs and practices in either moderating or increasing distress during times of adversity is linked to the individual’s beliefs regarding the nature of God and the individual’s sense of closeness to God (22, 56, 57, 132). In a meta-analysis of the impact of prayer on health, for example, Masters and Spielmans (57) found that the impact of prayer frequency on mental health outcomes was related to the study participants’ feelings of closeness to God. Those who felt subjectively close to God reported increased perceptions of wellbeing through their prayers, but greater loneliness, depression, and tension were felt when ritualistic prayers were combined with a sense of being distant from God (57). Another study on post-traumatic stress disorder (PTSD) among Muslim refugees affected by natural disasters in Central Sulawesi, Indonesia, found that coping was affected by the study participants’ “affinity to God” (56). The authors state that:

Individuals who had a stronger relationship with God were able to attribute more positive meaning to disaster and loss. Individuals who surrendered all matters to God were able to process the experience and develop acceptance (56, p. 61).

In mental health responses with Muslims affected by adversity, emphasis on the care and nearness of God may be particularly helpful. In their research, Bonab et al. (23) postulate that Islamic texts, Qur’anic stories, and “Allah’s 99 Beautiful Names,” such as The Preserver and Bestower (*Al-Mu’min*), The Responsive (*Al-Mujib*), The Ever Provider (*Al-Razzaq*), The Shielder (*Al-Mani’e*), and The Everlasting Refuge (*As-Samad*), point to attributes of Allah as an ideal attachment figure—one who is benevolent, willing, and able to help and near to the believer (23). The 99 Beautiful Names refer to the divine attributes of Allah as revealed in the Qur’an and highlighted in the Hadith (a collection teachings and sayings of the Prophet Mohammed). These names may be used as reflection and prayer tools by adherents to the Muslim faith, as they seek to establish or recover a sense of the benevolence, responsiveness, and care of Allah in their reappraisal process (134, 135).

### 5.3 Gender-blind approaches creating barriers to faith practice for women

The study also found evidence that poor gender sensitivity among some aid responders, camp management, and MHPSS providers, coupled with practitioners’ assumptions about the role of local faith leaders as the responsible parties for enabling access of women in the camp to faith-related supports, led to women being denied access to religious resources to which they are entitled in international human rights conventions (136–138) and humanitarian standards (35, 37, 65, 139, 140).

Specifically, UN-backed MHPSS guidelines along with industry-wide minimum standards call upon aid responders to “facilitate conditions for appropriate communal cultural, spiritual, and religious healing practices” (35, p. 106), provide inclusive spaces for religious practice in displacement camps (139), and include faith-sensitive considerations in programming across sectors (140). These directives appear to be minimally understood and applied in the camp where the study took place, however. There were indications in fact that key standards of practice are actively misunderstood and misapplied, namely, a misperception that the widely embraced ICRC Code of Conduct (141) for disaster relief responders *prohibits* responsiveness to faith priorities in emergency responses. In reality, the neutrality and impartiality mandates within the Code of Conduct prohibit *coercion* and selective aid provision in relation to religion (and other identities). That prohibition on coercion includes a prohibition against *excluding* support needs related to faith that are expressly stated as priorities in needs assessments, based on the belief system (secular or religious) of the responding organization.

The misconception among responders that formal aid actors (both local and international) should avoid facilitating access to appropriate coping supports adapted to faith and culture if requested, along with assumptions that the male imams would facilitate access to faith-related coping supports, resulted in the faith-related support needs of women not being systematically met by anyone. The deferral to the local male imam as responsible for such assistance demonstrated a poor understanding of gender norms in the context. Specifically, limited understanding among responders regarding the cultural norm that women should not communicate directly with men outside of their families meant that female-headed households that did not have a male relative to liaise with the imam on behalf of the household had less access to religious resources for coping than others in the camp.

This type of intersection between faith and gender creating access barriers has been observed in other research among displaced Muslim women (73, 76, 77, 142). Pertek et al. (142), reporting on analysis of interviews with 58 Muslim and Christian forced migrant women in Turkey, Sweden, the UK, and Australia who are survivors of gender-based violence (GBV), revealed a similar trend of gender and religion intersecting to heighten vulnerability. Women in that study likewise reported “barriers in accessing religious communal resources” in their displacement locations as a result of gendered discrimination rooted in the patriarchal power dynamics that are embedded in many religious structures. The authors call upon “professionals (working amongst displaced women of faith) to build capacities to prevent, mitigate and respond to such risks” (142, p. 12).

In a separate study among 36 Muslim female refugees, Shaw et al. (73) also found that gender and faith identities intersected with vulnerabilities related to their identity as migrants, leading the women who otherwise spoke of their faith as central to their coping to conceal their faith in public due to fears of persecution (73, p. 528). The researchers recommend that service providers working with forcibly displaced populations:

Recognize and draw upon the centrality of religiosity and gendered experiences when building casework, community, and mental health–related programs … Attention to respecting and enhancing religious and spiritual supports is key to promoting wellbeing among many communities of forced migrant women (73, p. 530).

## 6 Conclusion

This study has shown ways in which the faith-infused worldviews of displaced and conflict-affected Muslim women may influence fundamental understandings related to the source of internal struggles, the definition of wellbeing, and the pathways of distress alleviation. The study has also shown that the meanings attributed can be potent drivers of both comfort and distress and that that mental health responses that make space for persons of faith to reveal distress they may feel in relation to their faith and those that facilitate access to faith-coping supports prioritized (if any) by the individuals of faith can have transformative impacts on distress alleviation and perceived wellbeing.

Although facilitating access to faith-coping supports is already recommended broadly by international MHPSS guidelines for responses in emergency contexts (35, 37, 65), in light of the vital function of faith practices in relation to many Muslims conceptualization of wellbeing and the links demonstrated between self-appraisals of faith and distress levels, it is critical that in mental health responses among displaced Muslim women that barriers to faith practice are actively identified and removed and access is facilitated for all who desire such support. As detailed in the study, wellbeing was described by participants as a sense of comfort through connectedness to Allah now, with the aim of achieving eternal wellbeing, or union with Allah in Paradise following the Day of Judgment, when all Muslims will be judged according to the deeds of their life. Achieving eternal wellbeing was seen to be the ultimate priority of life by most, with this “Paradise Orientation” forming a key lens through which adversities in life were interpreted, daily decisions were made, and coping strategies were prioritized. Faith practices, including obligatory Islamic duties (such as the five pillars) and voluntary devotional practices (such as Sunnah practices), were seen as key pathways to coping and were seen to facilitate perceived growth toward that ultimate goal. These perceptions align with Islamic teaching regarding the pathways to wellbeing (43–54). Because of the importance of faith practice in regards to the wider “Paradise Orientation” of life, individuals’ self-appraisals of the “sufficiency” of their faith practice in fulfilling the faith-related practices commanded by the faith were strongly linked to distress outcomes.

Although the majority of respondents were able, over time, to mobilize many of the faith supports needed and there was a high level of faith practice, with prayer, reciting, reading, or listening to the Qur’an, and fasting widely reported as means of soothing painful emotions, reducing anxiety, and receiving provision and protection from God, self-appraised “inadequacy” of faith practice frequency was strongly linked with elevated distress. Self-appraised *decline* in faith post-conflict and displacement was described by the study participants with anguish and was strongly linked to mental health decline. Some of the reasons cited, however, for this decline were compounded by external barriers—lack of communal prayer space for women, mental health counselors avoiding or dismissing faith concerns, limited access to electricity to charge phones needed to be able to listen to the Qur’an by women with limited literacy, or limited access to the sacred text itself for women who were literate.

Thus, among displaced Muslim women, priority needs to be given to removing gender barriers and facilitating access to faith coping supports for those who desire these supports. Aid responders and MHPSS practitioners need to be trained in the gender-sensitive, appropriate application of the ICRC Code of Conduct (141) along with the means for making diverse sectoral and mental health supports responsive to faith considerations. In a camp context, faith-coping supports may include components such as spaces (including women’s only spaces when cultural norms require this) to gather with other women for peer-to-peer support or communal faith practice, religious materials like sacred texts (written and digital, including access to electricity and phone credit so that women who are illiterate may listen), and connection with respected persons of faith who can teach and console (including women of faith who are leaders but do not have formal titles). Such facilitation does not necessarily mean direct provision but identifying and connecting with the existing faith communities and leaders (male and female) in the context that may provide the required response.

When working with Muslim women who require focused or specialized mental healthcare—and wish to have faith engaged in their response—seeking to understand what role, if any, the individual’s beliefs regarding the afterlife and their perception of their position in relation to Paradise may be playing in distress alleviation (or elevation) may be important. Additionally, interventions that support the women to identify their own place within the unfolding, over-arching narrative of their faith and identify with founders or “heroes” of the faith who faced trials but ultimately prevailed may have therapeutic benefit. In light of the finding that the function of faith practices in distress alleviation was contingent on the security of the attachment to God (the individual’s perception regarding the nature of Allah’s care for, responsiveness to and nearness to the believer) and that for those who felt they had lost a prior sense of secure attachment, this was both measurably and descriptively associated with acute emotional distress—interventions that reinforce a sense of the benevolence and care of God may be beneficial. The “99 Beautiful Names” of Allah may be useful as reflection tools. Finally, given the finding that violence, particularly that which is perpetrated by groups like ISIS that have religious ideological elements against other religious believers, can severely test a believer’s perceptions of the nature of God and that a *persisting* fracture in a former sense of attachment to a benevolent universe can result in mental health decline, it is recommended that when the nature of traumatic experiences shared have religious elements, responders be alert to the way in which those experiences may amplify the vulnerability and distress of persons of faith.

Any consideration of inclusion of faith language, practices, or faith actors in responses, however, should be directed by the recipients of care. It is recommended that standardized questions are included in initial assessments. Being sensitive to faith in mental health responses includes protecting those of no faith and those of minority faith beliefs; thus, any adaptations considered need to be done on a case by case.

The findings from this study reinforce themes from the wider research community, including findings from many other studies that have found that positive religious coping is strongly associated with positive mental health benefits. This study is among the first, however, to highlight the powerful impact on distress levels of self-appraised faith sufficiency in light of the wider “Paradise Orientation” that serves as the lens of interpretation. This study also provides measurable links between perceptions of God’s care and self-appraisals of faith practice with mental health. In addition to the language barrier, one of the limitations of this study is that a preferred longitudinal approach to assess which faith practices were most associated with improvement, stability, or decline over time was prohibited by cost and access restrictions. Further mixed methods research is needed on the role of religious coping within other protracted displacement settings and on the functions of belief systems regarding the afterlife in buffering or elevating distress levels among Muslim populations. Similarly, studies on the relationship between religious coping and distress linked to daily stressors in displacement contexts is also warranted, with longitudinal studies recommended.

## Data Availability

The raw data supporting the conclusions of this article will be made available by the authors, without undue reservation.

## Footnotes

1 The five pillars include the profession of faith (*Al-Shahada—*that there is no God but Allah and that Mohammed is the Prophet of God), doing *Salat* (the five daily prayers*—*at dawn or before sunrise, when the sun is at its highest, in late afternoon, just after sunset and between sunset and midnight), fasting for the month of Ramadan (the ninth month of the lunar year during which fasting is required from dawn until sunset), giving of *zakat* (2.5% of one’s wealth in a lunar year, for religious and charitable purposes), and making a pilgrimage to Mecca in one’s lifetime (46–48).

2 “Occupation” is the term used by the women in the study.

3 See **Figure 1** for the three research questions outside of the scope if this article.

4 The HTQ, PMIR, and Kessler 10 were chosen from among more than 40 tools as the most appropriate for the context, validated among comparable populations and most aligned with the research questions. Select portions of the lengthy HTQ and PMIR tools (14 items from the HTQ and 3 subscales of the PMIR, respectively) were included in alignment with the guidelines for use of the tools to minimize the overall time burden on participants and to avoid potentially distressing questions that were not essential to the study.

5 The design is described as a “partial,” rather than “pure” explanatory sequential mixed methods approach, as the analysis of survey findings had not been completed prior to the start of the qualitative data collection. The “partial” nature refers to the daily, rapid real-time analysis of survey data, which informed the selection of the interview and FGD participants to be recruited the next day and directed related subject prioritization during those discussions.
