# Supplementary material for: Rethinking pathways to well-being: the function of faith practice in distress alleviation among displaced Muslim women affected by war
Source: Front Psychiatry. 2025 Jul 21;16:1335640. doi: 10.3389/fpsyt.2025.1335640 (PMC12320534; doi:10.3389/fpsyt.2025.1335640)
Supplement: Supplementary file 1 [file Table1.docx]

# Supplementary Material

# Appendix A Study Questionnaire with Translation

**PARTICIPANT NUMBER:__________**

رقم المشارك

**PARTICIPANT INFORMATION SHEET & TRAUMA EVENTS**

ورقة معلومات المشاركين والصدمات

***Complete First Time Only***

*التكملة للمرة الاولى فقط*

| ***This Section For Researcher Only:***  *هذا الفصل للباحث فقط* |  | | | | | | | | |
| --- | --- | --- | --- | --- | --- | --- | --- | --- | --- |
| ***Camp Name***  *اسم المخيم و رقم الخيمة ( ان امكن)* |  | | | | | | | | |
| ***Location of Interview***  *موقع المقابلة* | **In Tent/Home 1**  في الخيمة/ المنزل | | | | **At Centre 2**  في المركز | | | | **Other 3**  اخرى |
|  |  | | | |  | | | |  |
| ***Primary Language Used in Interview***  *اللغة الاساسية المستخدمة في المقابلة* | **Arabic 1**  اللغة العربية | | | **Kurdish 2**  اللغة الكردية | | | **English 3**  اللغة الانكليزية | | **Other 4**  اخرى |
|  |  | | |  | | |  | |  |
| ***Survey Date (Example: 14/03/2019)***  *تاريخ الاستبيان ( مثال: 14/3/2019)* | **Day**  يوم | | | | **Month**  شهر | | | | **Year**  سنة |
|  |  | | | |  | | | |  |
| **SECTION I: PERSONAL INFO & DEMOGRAPHICS**  القسم الاول: المعلومات الشخصية و المعلومات الديموقراطية |  | | | | | | | | |
| **WHAT IS YOUR AGE? (If not known list Unknown)**  ما هو عمرك؟(ان لم يكن معروفا سجل غير معروف) |  | | | | **If unknown, circle estimate:**  اذا غير معروف,ضع دائرة تقديريا:  **18-20 20-30 30-40 40-50 50-60 60+** | | | | |
| **RELATIONSHIP STATUS**  الحالة الاجتماعية | **Married (1)**  متزوجة | **Widow (2)**  ارملة | | | **Divorced**  **(3)**  مطلقة | | **Never Married**  **(4)**  لم تتزوج من قبل | | **Husband Missing**  **(5)**  الزوج مفقود |
|  |  |  | | |  | |  | |  |
| **ARE YOU FROM MOSUL ORIGINALLY?**  هل اصولك الفعلية من الموصل؟ | **YES (1)**  نعم | | | | | | **NO (2)**  لا | | |
|  |  | | | | | |  | | |
| **IF NOT FROM MOSUL, WHAT CITY FROM ORIGINALLY?**  **ا**ن لم تكن من الموصل, ماهي مدينتك الاصلية؟ |  | | | | | | | | |
| **IF NOT MOSUL, WHAT GOVERNORATE?**  ان لم تكن من الموصل, ماهي محافظتك؟ |  | | | | | | | | |
| **WHEN DID YOU LEAVE MOSUL or HOME LOCATION? (Month and Year: Example 03/2018)**  متى غادرت الموصل او االمنزل؟ (شهروسنة: مثال 3/2018) | **Month**  شهر | | | | | | **Year**  سنة | | |
|  |  | | | | | |  | | |
| **HAVE YOU BEEN DISPLACED SINCE THAT TIME?**  هل تم تهجيرك منذ ذلك الوقت؟ | **YES (1)**  نعم | | | | | | **NO (2)**  لا | | |
|  |  | | | | | |  | | |
| **HOW MANY TIMES HAVE YOU DISPLACED SINCE LEAVING MOSUL or HOME LOCATION?**  ماعدد المرات التي سبق لك فيها النزوح منذ ان غادرت الموصل او المنزل؟ |  | | | | | | | | |
| **HAVE YOU TRIED TO RETURN TO MOSUL (OR HOME LOCATION) AND THEN CAME TO THIS CAMP?**  هل حاولت العودة الى الموصل (او المنزل)وبعدها جئت الى هذا المخيم؟ | **YES (1)**  نعم | | | | | | **NO (2)**  لا | | |
|  |  | | | | | |  | | |
| ***If Yes, Why did you come back to the camp?***  *اذا كانت الاجابة بنعم, لماذا عدت الى المخيم؟* |  | | | | | | | | |
| **HOW MANY MONTHS HAVE YOU BEEN IN THIS**  **CAMP?**  ما عدد الشهور التي قضيتها في هذا المخيم؟ |  | | | | | | | | |
| **ARE YOU THE HEAD OF HOUSEHOLD (IN THE CAMP)**  هل انت رب الاسرة(في المخيم)؟ | **YES (1)**  نعم | | | | | | **NO (2)**  لا | | |
|  |  | | | | | |  | | |
| **IF YOU ARE NOT THE HEAD OF THE HOUSEHOLD, WHO IS: CHOOSE ONLY ONE**  **ا**ذا لم تكن انت رب الاسرة, فمن يكون : اختر واحد فقط | **Husband (1)**  الزوج | | **Father**  **(2)**  الاب | | | **Mother**  **(3)**  الام | | **Brother**  **(4)**  الاخ | **Grandfather**  **(5)**  الجد |
|  |  | |  | | |  | |  |  |
|  | **Sister**  **(6)**  الاخت | | **Son**  **(7)**  الابن | | | **Daughter**  **(8)**  الابنة | | **Uncle**  **(9)**  العم/ الخال | **Grandmother**  **(10)**  الجدة |
|  |  | |  | | |  | |  |  |
|  | **Aunt**  **(11)**  العمة/الخالة | | **Cousins**  **(12)**  ابن العم او الخال | | | **Brother-in-Law**  **(13)**  شقيق الزوج | | **Father-In-Law**  **(14)**  والد الزوج | **Mother-in-law**  **(15)**  والدة الزوج |
|  |  | |  | | |  | |  |  |
|  |  | |  | | |  | | **Other:**  **(17)**  اخرين | **Sister in Law**  **(16)**  اخت الزوج |
|  |  | |  | | |  | |  |  |
| **HOW MANY CHILDREN AGES 18 YRS & BELOW ARE LIVING WITH YOU?**  كم عدد الاطفال الذين يعيشون معك والتي تتراوح اعمارهم اقل من 18 عاما؟ |  | | | | | | | | |
| **WHAT OTHER RELATIONS ARE WITH YOU IN THIS CAMP? LIST ALL THAT APPLY**  كم عدد المعارف المتواجدين معك في نفس المخيم؟ اذكرهم جميعا | **Husband (1)**  الزوج | | **Father**  **(2)**  الاب | | | **Mother**  **(3)**  الام | | **Brother**  **(4)**  **ا**لاخ | **Grandfather**  **(5)**  الجد |
|  |  | |  | | |  | |  |  |
|  | **Sister**  **(6)**  الاخت | | **Son**  **(7)**  الابن | | | **Daughter**  **(8)**  الابنة | | **Uncle**  **(9)**  العم/الخال | **Grandmother**  **(10)**  الجدة |
|  |  | |  | | |  | |  |  |
|  | **Aunt**  **(11)**  العمة/الخالة | | **Cousins**  **(12)**  ابن العم او الخال | | | **Brother-in-Law**  **(13)**  شقيق الزوج | | **Father-In-Law**  **(14)**  والد الزوج | **Mother-in-law**  **(15)**  والدة الزوج |
|  |  | |  | | |  | |  |  |
|  |  | |  | | |  | | **OTHER**  **(12)**  اخرين | **Sister in Law**  **(16)**  **ا**خت الزوج |
|  |  | |  | | |  | |  |  |

**Section II: Trauma Events:**

**القسم الثاني: احداث الصدمة**

We would like to ask you about your past history. However, you may find some questions upsetting. If so, please feel free not to answer. Please feel free to stop the interview at any time.

نود ان نسألك عن تاريخك السابق. مع ذلك, سوف تجدين بعض الاسئلة المؤلمة. اذا كانت كذلك, لديك الحرية بعدم الاجابة.لا تتردد في ايقاف المقابلة في اي وقت.

Please indicate whether you have experienced any of the following events (**"**YES**"** or **"**NO**"** or “Prefer Not to Answer” for each column).

يرجى الاشارة الى ما اذا كنت قد واجهت ايا من الاحداث التالية ( " نعم " او " لا " او " تفضل عدم الاجابة " على اي سؤال في الجدول).

|  | **SECTION II: TRAUMA EVENTS**  القسم الثاني : احداث الصدمة | **PNTA=Prefer Not to Answer**  افضل عدم الاجابة | | |
| --- | --- | --- | --- | --- |
|  | | YES 1  نعم | NO 2  لا | PNTA 3  عدم الاجابة |
| **2A** | Oppressed because you were not religious enough?  مضطهد لانك لم تكن متدينا بما فيه الكفاية؟ |  |  |  |
| **2B** | Property stolen, confiscated, or destroyed  الممتلكات المسروقة, المصادرة ,او المدمرة |  |  |  |
| **2C** | Suffered poor health without access to medical care or medicine  عانيت من سوء الصحة دون الحصول على الرعاية الطبية او الدواء |  |  |  |
| **2D** | Suffered from lack of food or clean water  عانيت من نقص الطعام او المياه النظيفة |  |  |  |
| **2E** | Lacked shelter  افتقار للمأوى |  |  |  |
| **2F** | Witnessed the destruction of religious shrines or places of religious instruction  شهدت على تدنيس الاضرحة الدينية و الاماكن الدينية |  |  |  |
| **2G** | Exposed to combat situation (gunfire, explosions, artillery fire, shelling) or landmine  تعرضت لموقف قتال ( اطلاق نار, انفجارات, قصف مدفعي, الغام ارضية ) |  |  |  |
| **2H** | Experienced serious physical injury  تعرضتي لاصابة جسدية خطيرة |  |  |  |
| **2I** | Serious physical injury of family member or friend  تعرض احد افراد لعائلة او صديق لاصابة جسدية خطيرة |  |  |  |
| **2J** | Confined to home because of chaos and violence outside  اجبرت على البقاء في المنزل بسبب الفوضى والعنف في الخارج |  |  |  |
| **2K** | Disappearance of a family member (child, spouse, etc.)  اختفاء احد افراد الاسرة ( طفل , زوج , اخرين) |  |  |  |
| **2L** | Death of a family member  وفاة احد افراد الاسرة |  |  |  |
| **2M** | Death of friend  وفاة صديق |  |  |  |
| **2N** | Witnessed dead bodies or human remains  جثث الموتى او الاجسام المتبقيه |  |  |  |
| ***Please specify any other situation you would like to share (IF ANY):***  *يرجى تحديد اي موقف اخر ترغب بمشاركته ( ان وجد)* | | | | |
| ***Researcher Notes:***  *ملاحظات الباحث* | | | | |

**PARTICIPANT NUMBER: _______________**

**IRAQ WOMEN’S STUDY QUESTIONNAIRE:**

**III. MEASURE OF DISTRESS** Questions about anxiety and depressive symptoms that a person has experienced in the **MOST RECENT 4 WEEK PERIOD**.

أسئلة عن الأضطراب:

اسئلة عن علامات الاضطراب و الكابة التي واجها الشخص في الاسابيع الاربعة الاخيره

|  |  | **None of the time**  **ولا مره**  **(1)** | **A little of the time**  قليل من الاحيان  (2) | **Some of the time**  **بعض** **الاحيان**  **(3)** | **Most of the time**  **اغلب الأوقات**  **(4)** | **All of the time**  **كل الاوقات**  **(5)** |
| --- | --- | --- | --- | --- | --- | --- |
|  | **3A** In the last four weeks, about how often did you feel tired out for no good reason?  **كم مرة تقربيا شعرت بالتعب بدون سبب وجيه في الاسابيع الاربعة الاخيرة؟** |  |  |  |  |  |
|  | **3B** In the last four weeks, about how often did you feel nervous?  **كم مرة تقربيا شعرت بالتوتر في الاسابيع الاربعة الاخيرة؟** |  |  |  |  |  |
|  | **3C** In the last four weeks, about how often did you feel so nervous that nothing could calm you down?  **كم مرة تقربيا شعرت بالتوتر الشديد لدرجة لاشيئ بامكانه تهدئتك في الاسابيع الاربعة الاخيرة ؟** |  |  |  |  |  |
|  | **3D** In the last four weeks, about how often did you feel hopeless?  **كم مرة تقربيا شعرت بالياس في الاسابيع الاربعة الاخيرة؟** |  |  |  |  |  |
|  | **3E** In the last four weeks, about how often did you feel restless or fidgety?  **كم مرة تقربيا شعرت بالتوتر او القلق في الاسابيع الاربعة الاخيرة؟** |  |  |  |  |  |
|  | **3F** In the last four weeks, about how often did you feel so restless you could not sit still?  **كم مرة تقربيا شعرت بالقلق بحيث لا تستطيع الجلوس بهدوء في الاسابيع الاربعة الاخيرة؟** |  |  |  |  |  |
|  | **3G** In the last four weeks, about how often did you feel depressed?  **كم مرة تقربيا شعرت بالاكتاب في الاسابيع الاربعة الاخيرة؟** |  |  |  |  |  |
|  |  | **None of the time**  **ولا مره**  **(1)** | **A little of the time**  قليل من الاحيان  (2) | **Some of the time**  **بعض** **الاحيان**  **(3)** | **Most of the time**  **اغلب الأوقات**  **(4)** | **All of the time**  **كل الاوقات**  **(5)** |
|  | **3H** In the last four weeks, about how often did you feel that everything was an effort?  **كم مرة تقربيا شعرت بان كل شيء تفعله يتطلب منك بذل مجهود كبير في الاسابيع الاربعة الاخيرة؟** |  |  |  |  |  |
|  | **3I** In the last four weeks, about how often did you feel so sad that nothing could cheer you up?  **كم مرة تقربيا شعرت بالحزن الشديد وانه لا يوجد شيء يبهجك في الاسابيع الاربعة الاخيرة؟** |  |  |  |  |  |
|  | **3J** In the last four weeks, about how often did you feel your life was worthless?  **كم مرة تقربيا شعرت بانك عديم القيمة في الاسابيع الاربعة الاخيرة؟** |  |  |  |  |  |
|  | **3K** In the last four weeks, HOW MANY DAYS were you TOTALLY UNABLE to work, study or manage your day to day activities because of these feelings?  كم يوم تقريبا كنت غير قادر على العمل, الدراسة او تنظيم نشاطاتك اليومية بسبب هذا الشعور في الاسابيع الاربعة الماضية؟ | Number of days:  عدد الايام: | | | | |
|  | **3L** [Aside from those days], in the last 4 weeks, HOW MANY DAYS were you able to work or study or manage your day to day activities, but had to CUT DOWN on what you did because of these feelings?  (الى جانب هذه الايام) كم يوم تقريبا كنت قادرعلى العمل, الدراسة او تنظيم نشاطاتك اليومية ولكن اضطررت الى التوقف بسبب هذا الشعور في الاسابيع الاربعة الماضي | Number of days:  عدد الايام: | | | | |
|  | **3M** In the last 4 weeks, how many times have you seen a doctor or any other health professional about these feelings?  كم مرة قابلت دكتور او اي اخصائي صحي بسبب هذا الشعور في الاسابيع الاربعة الاخيرة؟ | Number of consultations:  عدد الأستشارات: | | | | |
|  | | **None of the time**  **ولا مرة**  **(1)** | **A little of the time**      **قليلا**  الاحيان  **(2)** | **Some of the time**  **(3)**  **بعض** ا**لأوقات**  **(3)** | **Most of the time**  **(4)**  **اغلب الأوقات**  **(4)** | **All of the time**  **(5)**  **كل الأوقات**  **(5)** |
| **14** | **3N** In the last 4 weeks, how often have physical health problems been the main cause of these feelings?  كم مرة كانت مشاكل الصحة البدنية المسبب الرئيسي لهذا الشعور في الاسابيع الاربعة الاخيرة؟ |  |  |  |  |  |
| ***Researcher Notes: (ملاحظات الباحث)*** | | | | | | |

**IV. MEASURE OF DISTRESS PART 2:** How much these symptoms have bothered a person **IN THE PAST ONE WEEK (7 days):**

أسئلة عن الأضطرابات الجزء الثاني:

كم ازعجت هذه الاعراض الشخص في الاسبوع الأخير (سبعة أيام):

|  |  | **Not at all**      **ولا مره**  **(1)** | **A little**  **(2)**  قليلا  *(2)* | **Quite a bit**  **(3)**  **الى حد كبير**  ***(3)*** | **Extremely**  **(4)**  **كثيرا**  **(4)** |
| --- | --- | --- | --- | --- | --- |
| **1.** | **4A** In the past one week, how often have you been bothered by Qalbak maqboud (sensation of the heart being squeezed)?  *كم مرة انزعجت من الشعور بان قلبك مقبوض في الاسبوع الماضي؟* |  |  |  |  |
| **2.** | **4B** In the past one week, how often have you been bothered by Nafseetak ta’bana (tired soul)?  *كم مرة انزعجت من الشعور بان نفسيتك متعبة في الاسبوع الماضي؟* |  |  |  |  |
| ***Researcher Notes: (ملاحظات الباحث)*** | | | | | |

**V. GLOBAL RELIGIOUSNESS SUBSCALE (PMIR) & VI. CHANGE IN RELIGIOUSNESS**

**التدين العالمي و التغيير في التدين:**

|  |  | **Very low**  **(1)**  جدا  (1) | **Low**  **(2)**  ضعيفة  (2) | **Average**  **(3)**  متوسطة  (3) | **High**  **(4)**  جيدة  (4) | **Very High**  **(5)**  جيدة جدا  (5) |
| --- | --- | --- | --- | --- | --- | --- |
| **1.** | **5A** Currently, how do you describe your diyana**?**  **كيف تصف تدينك حاليا؟** |  |  |  |  |  |
| **2.** | **6A** How would you describe your diyana **before** the crisis?  **كيف تصف تدينك قبل الأزمة؟** |  |  |  |  |  |
| *If your diyana (religiousness) has changed, why has it changed?*  *اذا تغيرت \| تأثرت ديانتك, ماذا تغير بها؟* | | | | | | |

**VII. PERCEPTION OF SUPPORT – RELIGIOUS**

**ادراك الدعم - الديني**

|  |  | **Not at all true**  غير صحيح  (1) | **Usually not true**  غير صحيح عادة  (2) | **Usually true**  صحيح عادة  (3) | **Very**  **true**  صحيح جدا  (4) | **Not applicable**  لاينطبق  (5) |
| --- | --- | --- | --- | --- | --- | --- |
| **1.** | **7A** I worry about religious issues.  *انا قلق بشأن مسائل دينية.* |  |  |  |  |  |
| **2.** | **7B** I feel that God cares about my life and situation.  أشعر بأن الله يعتني بحياتي و وضعي |  |  |  |  |  |
| **3.** | **7C** I feel close to Allah.  **أشعر بقربي من الله** |  |  |  |  |  |

**VIII. Islamic Duty Subscale (PMIR)**

**واجبات اسلامية**

|  |  | **Never**  **(1)**  ولا مرة  (1) | **A few times a year**  **(2)**  مرات قليلة في العام  (2) | **Several times a month**  **(3)**  مرات عديدة  (3) | **Several times a week**  **(4)**  مرات عديدة في الاسبوع  (4) | **Most of the time the five daily prayers**  **(5)**  اغلب الاوقات خمس مرات باليوم  (5) | **Five times a day or more**  **(6)**  حمسة مرات في اليوم او اكثر  (6) |
| --- | --- | --- | --- | --- | --- | --- | --- |
|  | **8A** How often do you pray?  كم مرة تصلي؟ |  |  |  |  |  |  |
|  |  | **Never**  **(1)**  ولا مرة  (1) | **A few times in life**  **(2)**  مرات قليلة بالحياة  (2) | **Few days of the month of Ramadan each year**  **(3)**  ايام قليلة من شهر رمضان كل عام  (3) | **Half to All the month of Ramadan each year**  **(4)**  نصف الى كل شهر رمضان من كل عام  (4) | **The whole month of Ramadan each year**  **(5)**  كل شهر رمضان في كل عام  (5) | **Other religious days or sunnah fasts in addition to Ramadan (6)**  ايام دينية او سنة الأثنين و الخميس بالأضافة الى رمضان |
|  | **8B** How often do you fast?  كم مرة تصوم؟ |  |  |  |  |  |  |

|  |  | **Never**  **(1)**  ولا مرة  (1) | **A few times in life**  **(2)**  مرات قليلة  (2) | **A few times a year**  **(3)**  مرات قليلة في العام  (4) | **A few times a month**  **(4)**  مرات قليلة بالشهر  (4) | **About once or twice a week**  **(5)**  تقريبا مرة او مرتان بالاسبوع  (5) | **Once a day or more**  **(6)**  مره او اكثر باليوم  (6) |
| --- | --- | --- | --- | --- | --- | --- | --- |
|  | **8C** How often do you go to the masjid?  كم مرة تذهب للمسجد؟ |  |  |  |  |  |  |
|  | *If there was a mosque (for women), would you like to go?*  **اذا كان هناك مسجد هل تحب ان تذهب ؟** | | | | | | |
|  | **8D** Except in prayers, how often do you read or listen to the Holy Qura’n?  كم مرة تقرأ او تستمع للقران الكريم ماعدا اثناء الصلاة؟ |  |  |  |  |  |  |
|  | **8E** Except in prayers, how often do you engage in d’iker or tasbih?  كم مرة تنشغل بالذكر اوالتسبيح ماعدا اثناء الصلاة؟ |  |  |  |  |  |  |

**IX. Islamic Positive Religious Coping Subscale & X. Punishing Allah Reappraisal Subscale**

**التأقلم الديني الاسلامي الايجابي و اعادة التقييم والعقاب**

|  |  | **I do not do this at all**  **لا افعل هذا ابدا**  **(1)** | **I do this a little**  **افعل هذا قليلا**  **(2)** | **I do this a medium amount**  **(3)**  ب**شكل متوسط**  **(3)** | **I do this a lot**  **(4)**  **كثيرا**  **(4)** |
| --- | --- | --- | --- | --- | --- |
| **1.** | **9A** When I face a problem in life, I look for a stronger connection with Allah  عند مواجهة مشكلة بالحياة, اتقرب اكثر من الله |  |  |  |  |
| **2.** | **10A** When I face a problem in life, I believe that I am being punished by Allah for bad actions I did  عندما تواجهني مشكلة في الحياة, اتصور بأن هذا عقاب من الله بسبب ارتكابي أفعال سيئة |  |  |  |  |
|  |  | **I do not do this at all**  **لا افعل هذا ابدا**  **(1)** | **I do this a little**  **افعل هذا قليلا**  **(2)** | **I do this a medium amount**  **(3)**  ب**شكل متوسط**  **(3)** | **I do this a lot**  **(4)**  **كثيرا**  **(4)** |
| **3.** | **9B** When I face a problem in life, I consider that a test from Allah to deepen my belief  عندما تواجهني مشكلة في الحياة, اعتبر هذا اختبار من الله لتعميق ايماني. |  |  |  |  |
| **4.** | **10C** When I face a problem in life, I feel punished by Allah for my lack of devotion  عندما تواجهني مشكلة في الحياة, اتصور بأن هذا عقاب من الله بسبب عدم اخلاصي لله. |  |  |  |  |
| **5.** | **9D** When I face a problem in life, I read the Holy Qura’n to find consolation  عندما تواجهني مشكلة في الحياة, اتوجه لقراءة القران بحثا عن المواساة. |  |  |  |  |
| **6.** | **9E** When I face a problem in life, I ask for Allah’s forgiveness  عندما تواجهني مشكلة في الحياة, اطلب العفو من الله |  |  |  |  |
| **9.** | **10B** When I face a problem in life, I wonder what I did for Allah to punish me  عندما تواجهني مشكلة في الحياة, اتسائل ما الذي فعلته ليعاقبني الله |  |  |  |  |
| **10.** | **9C** When I face a problem in life, I seek Allah’s love and care  عندما تواجهني مشكلة في الحياة, اطلب الحب والعطف من الله |  |  |  |  |
| **11.** | **9F** When I face a problem in life, I remind myself that Allah commanded me to be patient  عندما تواجهني مشكلة في الحياة, اذكر نفسي بان الله امرني ان اكون صبورا. |  |  |  |  |
| **12.** | **9G** When I face a problem in life, I do what I can and put the rest in Allah’s hands  عندما تواجهني مشكلة في الحياة, اعمل مابوسعي واترك الباقي بيد الله. |  |  |  |  |
| ***Researcher Notes: (ملاحظات الباحث)*** | | | | | |

**XI. Other Beliefs:**

**معتقدات اخرى**

|  |  | **Yes**  **(1)**  **نعم**  **(1)** | **No**  **(2)**  **لا**  **(2)** | **Uncertain**  **(3)**  **غير متأكد**  **(3)** |
| --- | --- | --- | --- | --- |
| **1.** | **11A** I believe in the existence of the angels, the Jinn, and Satan  اؤمن بوجود الملائكة, الجن و الشيطان. |  |  |  |
| **2.** | **11B** I believe Jinn or Satan are causing problems in my life now.  اعتقد بأن الجن والشياطين هم من يسبب المشاكل في حياتي الان |  |  |  |

**XII. PERCEPTION OF SUPPORT – COMMUNITY**

**معرفة الدعم - المجتمع**

|  |  | **Yes**  **(1)**  نعم  (1) | **No**  **(2)**  لا  (2) | **Uncertain**  **(3)**  غير متأكد  (3) |
| --- | --- | --- | --- | --- |
| **1.** | **12A** I speak to family members to help me feel better about my situation.  اتحدث مع افراد الأسرة لمواساتي في وضعي |  |  |  |
| **2.** | **12B** I speak to other women in the community to feel better about my situation.  اتحدث مع نساء اخريات في المجتمع لمواساتي في وضعي |  |  |  |
| **3.** | **12C** I speak to religious leaders about my situation.  اتحدث مع رجال الدين لمواساتي في وضعي |  |  |  |
| *If they answer no, that they don’t speak to religious leaders, ask why they don’t speak to religious leaders?*  *اذا كان الجواب لا اتحدث مع رجال الدين,ما السبب الذي يمنعك من ذلك؟* | | | | |
| ***Other Researcher Notes: (ملاحظات اخرى للباحث)*** | | | | |

**XIII. PERCEPTION OF SUPPORT – AID WORK SPECIFIC**

**معرفة الدعم – خاص ب عمل المساعدات**

|  |  | **Yes (1)**  **نعم**  **(1)** | **No**  **(2)**  **لا**  **(2)** | **Uncertain**  **(3)**  **غير متأكد**  **(3)** |
| --- | --- | --- | --- | --- |
| **1.** | **13A** I feel that aid workers respect my religion.  اشعر بان عمال المساعدة يحترمون ديني |  |  |  |
| *If you do NOT feel that aid workers respect your religion, why not?*  *ان كنت تعتقد بان عمال المساعدة لا يحترمون ديانتك ,لم لا؟* | | | | |
| **2.** | **13B** When I first arrived in the camp, I had everything I wanted to practice my religion.  **حال وصولي الى المخيم, كنت امتلك كل مااحتاجه لممارسة فرائض الدين** |  |  |  |
| *If you felt you did NOT have everything you needed to practice your religion when you arrived in the camp, what was it you did not have that you wanted?*  *ان كنت تشعر بأنه لم تكن تملك كل شئ تحتاجه لكي تمارس ديانتك عندما وصلت الى المخيم ,ماالشئ الذي كنت تريده ولم تملكه؟*  *Since that time, have you received the item/support you wanted?*  *حتى هذا الوقت ,هل استلمت كل المواد والدعم الذي أردته؟*   - *IF YES, who provided that and how long did it take to receive it?* - *اذا نعم,من وفر هذا وكم من الوقت احتاج من اجل التسليم؟* - *IF NO, have you requested that support? From whom and when?* - *اذا لا,هل طلبت هذا الدعم؟من من ومتى؟* | | | | |
| **3.** | **13C** Now, I have everything I want to practice my religion.  **امتلك الان كل مااحتاجه لممارسة فرائض الدين** |  |  |  |
| *If you feel you do NOT have everything needed to practice religion, what is your concern?*  *اذا كانت غير مرضيه أو لاتمتلك كل ما تحتاج اليه لممارسة فرائض الدين, ماذا يقلقك؟* | | | | |
| **REFERRAL INFORMATION GIVEN TO PARTICIPANT: (Circle One)**  **معلومات الاحاله المعطاة للمشترك؟ نعم YES**  لا**NO**  ***For Researchers: at the end of the survey, provide the printed information with the referral details for the (Agency Confidential) Case Manager (Name Confidential) at (Phone Number Confidential).^^[[1]](#footnote-1)^^ Invite the participant to contact her if they would like to discuss any of the issues further.*** | | | | |
| ***Other Researcher Notes: (ملاحظات اخرى للباحث)*** | | | | |

# Appendix B Semi-Structured Interview Guide

**Summary Overview:**

Thank you for participating in the questionnaire. We would like to speak with you further if you permit. Your questionnaire information was very helpful, and we would like to hear more of your thoughts. The interview discussion will take approximately one hour.

**Just to reaffirm what we discussed before, similar to the questionnaire:**

- Participation in the interview is completely voluntary

المشاركة اختياريه تماما

- Your participation will not be discussed with others outside of this room. It will be completely private.

**مشاركتك في هذه الاستبيان لن تناقش مع الاخرين خارج هذه الغرفة .سوف تكون سرية للغاية..**

- You can skip topics and questions if you would not like to answer.

تستطيع تخطي الأجوبة في حال لم تحب الاجابة.

- IF you decide to take part today - you are still free to stop the interview at any time and you do not have to explain why.

اذا قررت ان تكون جزءا اليوم-سوف تبقى حرا للانسحاب خلال المقابلة او بعد ذالك ولست ملزما للتوضيح.

**Recording**

التسجيل(للمقابلات)

Also, as we discussed before, we would like to make a temporary recording of this interview. The recording is for translation and documentation only. The recording will be **deleted** after it is translated. It will only be available to Kathleen and the research assistant for a short-time. It will not be available to anyone else. Is this acceptable to you?

.لا نحتاج لتسجيل محادثة الاستبيان.اذا كنت مستمتع للمشاركة في مقابلة المناقشة المنفصلة.نود تسجيل مؤقت في الوقت الحاضر.التسجيل من اجل الترجمة وتوثيق البيانات.سوف تحذف التسجيلات بعد الترجمة ولن تكون متوفرة لأي شخص اخر.

**Topics of Discussion**

مواضيع النقاش

The topic of the discussion may be difficult.

موضوع النقاش قد يكون صعبا.

- We will ask about some of the events you have experienced in the past few years and now in your current situation.

سوف نسأل عن بعض الاحداث التي واجهتك في الموصل ومنذ وصولك التي قد تكون محزنة للغاية.

- We will also talk about religious beliefs.

سوف نتحدث ايضا حول المعتقدات الدينية.

- The purpose of this study is to look at the different ways in which you solve your problems.

الغرض من هذه الدراسة هو النظر الى الطرق المختلفة التي يتم من خلالها حل المشاكل .نحن خصيصا نود أن نسألكم عن تجاربكم في السنوات القليلة الماضية ,

**Questions and Confirmation:**

عملية الموافقة(الحرفية)

Do you have any questions? Is there anything you would like for us to explain again?

هل لديك أي سؤال ؟هل هناك أي شئ تود أن يوضح مره ثانية؟

Based on what you have heard, are you still in agreement to continue with the interview?

اعتمادا على ما سمعت ,هل توافق أ تشارك في الدراسة(والمقابلة)؟

**Introduction/Check-In:**

- You’ve answered many difficult questions regarding distressing events when we spoke before, how are you feeling?
- Is there anything more that you would like to share regarding your experience?

**BEFORE:**

- Looking back at 2014 and before the recent events, what was life like for you then? How did you spend your days?
- When you faced challenging things, how did you cope?

**DURING**

- In the time from when the events started until you left Mosul, what would you say your main concerns in life were?
- Was there anything that helped you feel better in that time?

**IN THE FIRST FEW MONTHS AFTER DISPLACING:**

- In the first few months when you were here, what were the things that **concerned** you most?
- Was there anything that helped you feel better at that point? What types of things?

**NOW:**

- In terms of life and stresses, how are you feeling now vs. when you first arrived in the camp?
  - If improvement: What types of *actions* have contributed to that improvement?
- When you think of the events that have happened, and the current situation, do you spend time thinking about why it has happened?
  - Why do you think it has happened/is happening?
  - Is this something that causes you distress in your life? If so, how does it affect you?
  - Note for researcher: If YES to jinn on the survey…ask for the person to describe how they feel jinn impacts their lives? And how they deal with this?
- If you have those thoughts, have those increased over time since you arrived or decreased or stayed the same? What, if anything, has helped you regarding those questions?
- Before the recent events, how would you describe your faith?
- Thinking about it now, how would you describe your faith at this point?
- Have your *beliefs* related to your faith and religion changed from before? If yes, how so?
- Have your *actions* related to faith changed from before?
  - IF YES, how have they changed?
  - Why do you think they have changed?
  - IF NO, what do you think has helped you most to maintain the same beliefs and practice? How has it helped?
- Presently, do you think about religious worries? If so, what types of things are on your mind?
- Since you have been here, at any point, did you wish for support to practice your religion?
  - IF YES, what did you think about?
  - IF YES, who do you think should support you (if anyone)?
  - IF YES: in the time since you arrived until now, at what point did you start to think of this things?
- Presently, do you think about religious support you would like?
  - If YES, what is on your mind?
  - IF YES, have you spoken to anyone about wanting this support? (If they say they have not – ask further – why they have not?).

**PRACTICALITIES:**

- What has your experience been like with aid workers? Specific examples?
- Do you feel that aid workers respect your faith and religious needs? Why or why not? Any specific examples?
- On practical support, is there anything specific you still desire in order to practice our religion? Who should provide that support?

**FUTURE**

- For future aid responses, if others are facing similar things, what type of support do you think people should have when they first arrive in a camp? What about after many years?

Do you have any questions for me?

***Note for Researcher:***

Referral Info for the Participant: Thank them for their participation. Acknowledge that the questions may have been difficult and explain that support is available if they would like to talk about these issues further. Give the referral card.

# Appendix C Information Sheet for Participants and Consent Form

**Participant Number:**

**:**

رقم المشترك

**------------------------------------------------------------------------------------------------**

**Information Sheet for Participants & Consent Form:**

**ورقة المعلومات للمشاركين &استمارة الموافقة:**

**Introduce Yourself to Participant**

**عرف نفسك للمشترك**

**Overview:**

**نظرة عامه:**

You have been invited to be involved in this study. Participating is completely voluntary. Before you decide whether you want to take part, we want to explain the project and what your participation will involve.

لقد دعوت من اجل المشاركة في هذه الدراسة .المشاركة اختيارية تماما .قبل ان تقرر فيما اذا اردت ان تكون جزءا ,نريد أن نوضح المشروع ومشاركتك سوف ماذا تتضمن .

**What is the Purpose of the Study?**

ما هو الغرض من هذه الدراسة؟

The purpose of this study is to look at the different ways in which you solve your problems. We particularly want to ask you about your experiences in the past few years

الغرض من هذه الدراسة هو النظر الى الطرق المختلفة التي يتم من خلالها حل المشاكل .نحن خصيصا نود أن نسألكم عن تجاربكم في السنوات القليلة الماضية ,

**Who is Carrying out the Research?**

من الذي يقوم بالبحث؟

The research is from Queen Margaret University in the UK. It is being led by a student called Kathleen. I am a research assistant with the project.

البحث هو مبادرة من جامعة مارغريت في المملكة المتحدة .التي تؤديها طالبه في الجامعة .هذه الطالبة اسمها :كاثلين .لقد عملت مع الناس الذين تأثروا بالنزاع الأخير .وأنا مساعدة بحث في هذا المشروع.

**Privacy**

السرية

Your participation in the survey will not be discussed with others outside of this room. It will be completely private.

**مشاركتك في هذه الاستبيان لن تناقش مع الاخرين خارج هذه الغرفة .سوف تكون سرية للغاية..**

**Not Related to Services or Aid Benefits:**

غير متعلق بتوفير الخدمات او المساعدات

The research is for learning only. It isn’t related to what you receive in the camp. Whether or not you participate won’t affect that at all. The results will help us learn and improve for the future.

البحث لا علاقه له بأي مساعده اخرى .فيما اذا شاركت او لا هذا لن يؤثر على الدعم الذي سوف تتلقاه من وكالات المساعدة النتائج. سوف تستعمل للتعلم فقط ومساعدة الناس بالمستقبل.

**Why have I been chosen?**

لماذا تم اختياري؟

We have asked you to be part of this study because you lived in Mosul during the recent difficulties.

لقد اخترناك لتكون جزءا من هذا البحث لأنك عشت في الموصل خلال الصعوبات الاخيرة ..

**What will participation involve?**

**ماذا سوف تتضمن المشاركة؟**

We will ask you to take a survey today. And we may like to visit a second time for an interview.

**سوف نطلب منك المشاركة في الاستبيان اليوم . وهناك احتمالية زيارة ثانية للمقابلة.**

- The total time required for the survey today will be approximately 1 hour.

الوقت الكلي المطلوب للدراسة اليوم سوف يكون تقريبا ساعه واحده.

- If we have a second visit, that will take approximately 1 hour also.

هناك مقابلة ثانية ,,سوف يأخذ هذا ما يقارب ساعة الواحدة ايضا.

- The questions in the survey only require yes or no answers, or selecting one of several answers on a list.

الأسئلة في الدراسة تتطلب فقط الإجابة بنعم أو لا ,او اختيار اجابه من اجابات عديده على القائمة.

- You can skip questions as well if you would not like to answer.

تستطيع تخطي الأجوبة في حال لم تحب الاجابة.

**Topics of Discussion**

مواضيع النقاش

The topic of the discussion may be difficult.

موضوع النقاش قد يكون صعبا.

- We will ask about some of the events you have experienced in Mosul and since you arrived that may have been very distressing.

سوف نسأل عن بعض الاحداث التي واجهتك في الموصل ومنذ وصولك التي قد تكون محزنة للغاية.

- We will also talk about religious beliefs.

سوف نتحدث ايضا حول المعتقدات الدينية.

- You can skip any questions you would like not to answer.

تستطيع ان تتخطى أي سؤال لا تريد الاجابة عليه

- You can also stop the interview at any time and we will not ask why.

تستطيع ايضا ان توقف المقابلة في أي وقت ولن نسأل عن السبب.

**Confidentiality & Anonymity**

**ا**لسرية &عدم كشف الهوية

We are hoping to return to the camp in six months time. If you are interested, we may want to meet with you again. We do need to ask for your Tent number and last name only. **This information will not be shared with others at any time**. The information will be kept with Kathleen only and it will be deleted at the end of the project. .

نحن نأمل بالعودة الى المخيم في الاشهر الستة القادمة .في حال كنت راغب نحن نود اللقاء بك ,نريد السؤال عن رقم الخيمة والاسم الاخير فقط .هذه المعلومات لن تشارك مع الاخرين في أي وقت .المعلومات سوف تكون محفوظة عند كاثلين فقط وسوف تقوم بحذفها في نهاية المشروع .

**Recording (For Interviews)**

التسجيل(للمقابلات)

We do not need to record this questionnaire conversation. If you are interested in participating in the separate interview discussion, we would like to make a temporary recording at that time. The recording is for translation and documentation only. The recording will be **deleted** after it is translated. It will not be available to anyone else.

.لا نحتاج لتسجيل محادثة الاستبيان.اذا كنت مستمتع للمشاركة في مقابلة المناقشة المنفصلة.نود تسجيل مؤقت في الوقت الحاضر.التسجيل من اجل الترجمة وتوثيق البيانات.سوف تحذف التسجيلات بعد الترجمة ولن تكون متوفرة لأي شخص اخر.

**Withdrawal from the study**

**ا**لانسحاب من الدراسة

Please note again that:

رجاءاً لاحظ مره اخرى:

- Participation is completely voluntary

المشاركة اختياريه تماما

- IF you decide to take part today - you are still free to withdraw during the interview or later and you do not have to explain why.

اذا قررت ان تكون جزءا اليوم-سوف تبقى حرا للانسحاب خلال المقابلة او بعد ذالك ولست ملزما للتوضيح.

**Consent Process (Verbal)**

عملية الموافقة(الحرفية)

Do you have any questions? Is there anything you would like for us to explain again?

هل لديك أي سؤال ؟هل هناك أي شئ تود أن يوضح مره ثانية؟

Based on what you have heard, do you agree to participate in the survey (and interview)?

اعتمادا على ما سمعت ,هل توافق أ تشارك في الدراسة(والمقابلة)؟

|  |
| --- |

No

لا

|  |
| --- |

Yes

نعم

**If NO, leave form blank**…. ملحوظة الباحث :في حال الاجابة ب لا ,اترك الاستمارة فارغة....

**Printed Name and Signature of researcher verifying response:**

الاسم المطبوع و توقيع الباحث للتحقق من الاستجابة:

__________________________________________________

**IF they have given consent please ask for the details below:**

***ملحوظة الباحث :اذا تمت الموافقة-من فضلك اسأل عن التفاصيل في الأسفل ..***

**TENT NUMBER (Arabic and English): _______________________**

**SURNAME (Arabic):________________________________________________**

الاسم الاول &الاخير (بالعربي)

**SURNAME (English):_______________________________________________**

اسم المشترك(بالإنكليزية)

**Date:_________________**

التاريخ :

***_Where do you prefer to make the survey?***

***أين ترغب بأجراء الدراسة؟***

***_When do you like (the interview) to be?***

***متى تحب أن تكون؟***

# Appendix D Research Team Code of Conduct

**CODE OF CONDUCT FOR RESEARCHER, RESEARCH ASSISTANTS AND INTERPRETERS**

**Research study on association between religious coping and recovery among Iraqi Muslim women affected by war and displacement**

To maintain the highest standards of conduct and integrity in this research we will adhere to the following Code of Conduct.

**Introduction**

Any code of conduct is built on a foundation of shared values. The values of this project include:

- All children and adults are of equal value;
- Inclusiveness and social justice;
- Respect for pluralism and diversity;
- Transparency, integrity and honesty;
- Responsible stewardship of resources; and,
- Commitment to carrying out our responsibilities to the best of our ability.

These values have informed the development of the Code of Conduct. The Code of Conduct should help guide your behaviour whilst engaged on this research project. However, because it is not possible to set forth an all-inclusive or exhaustive Code of Conduct, you are expected to use your own good judgment to conform to the intent and spirit of The Code in all matters not specifically addressed.

**A. Personal Conduct and Professional Integrity**

Members of the research team must act with honesty, integrity and openness in all their dealings as representatives of the project. You should:

- Treat colleagues and participants professionally and with courtesy and respect;
- Avoid any action which may be viewed as harassment, verbal or physical abuse;
- Avoid behaviour that may create an atmosphere of hostility or intimidation;
- Do not engage in any illegal activity;

**B. Confidentiality and disclosure of information**

- Ensure that participants give informed consent to their involvement in the assessment
- All information collected in the course of your work must be safeguarded, and must not be shared with anybody outside the project.
- Do not make copies of any of the data, or keep any record other than those required for the research project.
- Do not use confidential information to attempt to identify or make unauthorised contact with any individual, or provide information to another person for those purposes.
- Observe confidentiality procedures, including in casual conversation with others in the research team.

**Signature and Acknowledgment**

I certify that I have read and understand the Code of Conduct and agree to comply with it,

Printed Name: ____________________________

Signature: ____________________________ Date: ________________________

1. Referral details removed for thesis annexing. Details were provided on the field documents for researchers. The Arabic translation was also deleted during annexing for confidentiality of referral contact details. [↑](#footnote-ref-1)
